# The role of host promiscuity in the invasion process of a seaweed holobiont

**DOI:** 10.1038/s41396-020-00878-7

**Published:** 2021-01-21

**Authors:** Guido Bonthond, Till Bayer, Stacy A. Krueger-Hadfield, Nadja Stärck, Gaoge Wang, Masahiro Nakaoka, Sven Künzel, Florian Weinberger

**Affiliations:** 1 GEOMAR Helmholtz Centre for Ocean Research Kiel, Düsternbrooker Weg 20, 24105 Kiel, Germany; 2 Department of Biology, University of Alabama at Birmingham, 1300 University Blvd, CH464, Birmingham, AL 35294 USA; 3 College of Marine Life Sciences and Institute of Evolution and Marine Biodiversity, Ocean University of China, 5 Yushan Road, Qingdao, 266003 China; 4 Akkeshi Marine Station, Field Science Center for Northern Biosphere, Hokkaido University, Aikappu 1, Akkeshi, Hokkaido, 088-1113 Japan; 5 Max Planck Institute for Evolutionary Biology, August-Thienemann-Straße 2, Plön, 24306 Germany

**Keywords:** Symbiosis, Molecular ecology, Microbial ecology

## Abstract

Invasive species are co-introduced with microbiota from their native range and also interact with microbiota found in the novel environment to which they are introduced. Host flexibility toward microbiota, or *host promiscuity*, is an important trait underlying terrestrial plant invasions. To test whether *host promiscuity* may be important in macroalgal invasions, we experimentally simulated an invasion in a common garden setting, using the widespread invasive macroalga *Agarophyton vermiculophyllum* as a model invasive seaweed holobiont. After disturbing the microbiota of individuals from native and non-native populations with antibiotics, we monitored the microbial succession trajectories in the presence of a new source of microbes. Microbial communities were strongly impacted by the treatment and changed compositionally and in terms of diversity but recovered functionally by the end of the experiment in most respects. Beta-diversity in disturbed holobionts strongly decreased, indicating that different populations configure more similar –or more common– microbial communities when exposed to the same conditions. This decline in beta-diversity occurred not only more rapidly, but was also more pronounced in non-native populations, while individuals from native populations retained communities more similar to those observed in the field. This study demonstrates that microbial communities of non-native *A. vermiculophyllum* are more flexibly adjusted to the environment and suggests that an intraspecific increase in host promiscuity has promoted the invasion process of *A. vermiculophyllum*. This phenomenon may be important among invasive macroalgal holobionts in general.

## Introduction

Biological invasions have profound ecosystem impacts across the planet and represent one of the greatest threats to biodiversity [1–3]. How species become invasive and which traits and processes mediate biological invasions are key questions to address in order to develop strategies to prevent, intercept, and manage invasive species. In plant invasions, the manner in which hosts interact with microbiota in the environment, in particular the soil, is a determinant during invasion [4]. These interactions have been studied among invasive terrestrial plants, but less is known about invasive macroalgae in near shore marine ecosystems [5, 6]. Instead of microbes in the soil, macroalgae are in permanent interaction with microbiota in the water [7]. Similar to invasive plants, the ways in which macroalgal hosts engage in these interactions is likely fundamental to successful establishment in novel habitats. Seaweed holobionts (definition in [8]) are colonized by epi- and endophytic microbes. To these microbial communities, the hosting organism is more than simply a substrate, as it actively manipulates the associated microbiota to maintain a benign or even beneficial community. Hosts may do this, for instance, by producing compounds that attract or deter specific bacterial taxa, (e.g., [9–11]), inhibit quorum sensing (e.g., [12, 13]), stimulate the activity of specific bacterial taxa (e.g., [14]), or alter nutrient concentrations at the microscale to favor the proliferation of certain functional groups (e.g., [15]). Such host-mediated mechanisms facilitate a degree of control over the associated microbial community and are therefore essential traits, directly linked to performance and important predictors of the abundance and geographic distribution of the host itself [16].

While introductions are mediated by anthropogenic processes, they are typically unintentional and represent temporary or long-lasting extreme conditions that most species do not survive [17, 18]. Putatively, such extreme conditions rigorously disturb the associated microbial communities and once an invasive species is introduced, the new environment itself presents a second barrier. In order to successfully establish, invasive holobionts thus require a capacity to either protect benign or beneficial microbiota or to reconfigure them under new conditions. Different strategies may facilitate plants and macroalgae to become successful invaders. One such strategy, also known as the *accompanying mutualist hypothesis*, involves the co-introduction of microbial symbionts with specific functions, giving the invader a unique advantage in foreign habitats [19, 20]. Invasive legumes and actinorhizal plants, for instance, benefit from mutualistic relationships with tightly associated diazotrophic bacteria (e.g., [1]). Invasive holobionts may, however, also profit from being less dependent on specific taxa [21], which is postulated by the *generalist host hypothesis* [22]. Where the *accompanied host* requires specific microbiota and the success of its invasion thus depends on the co-introduction of these symbionts, the *generalist host* is more flexible – or more *promiscuou*s [23] – and can perform well while associating a wider range of microbes in a wider range of environments. Therefore, the *generalist host hypothesis* predicts that host promiscuity promotes invasiveness [20].

The red macroalga *Agarophyton vermiculophyllum* (Ohmi) Gurgel et al. is a widespread invader (see [24] and references therein) and is capable of manipulating associated microbial communities to its benefit [11, 25]. Several studies have demonstrated that the interaction between this host and associated microbiota differs between native and non-native populations [25–27]. Moreover, the microbial communities associated with *A. vermiculophyllum* vary in composition and function, across the scale of its known distribution, both locally and between the native and non-native range [28]. Together these studies suggest a shift in the interaction between host and microbiota may have occurred during the invasion process. Hypothetically, the holobiont disturbance during extreme transportation conditions and exposure to new microbial pressures in non-native environments could have acted as a selective filter for hosts with more promiscuous phenotypes.

If this is true, the more promiscuous non-native populations are likely more capable of configuring functional communities following stressful conditions similar to those experienced in the course of an introduction event. In other words, an introduction process would be less stressful to these promiscuous phenotypes. Combined with the Anna Karenina Principle – which predicts that microbiota of replicated holobionts disperse under stressful conditions (i.e., beta-diversity increases, [29]) – the generalist host hypothesis implies that when transplanted to a common garden, microbiota of native holobionts will disperse more than the microbiota of the more promiscuous (and therefore more invasive) non-native holobionts.

Here, we simulated an invasion with specimens originating from native and non-native *A. vermiculophyllum* populations in a common garden environment created under controlled conditions in the lab. After applying a holobiont disturbance treatment, we monitored succession trajectories of the associated microbial communities for six weeks. To test the implementation of the generalist host hypothesis that predicts that non-native *A. vermiculophyllum* holobionts are more promiscuous and more invasive, we formulated three sub-hypotheses: Following holobiont disturbance and introduction to the common garden (i) non-native holobionts perform better, (ii) non-native host associated communities from different populations become more similar toward each other (i.e., they configure a more common community) and (iii) microbial communities of non-native holobionts undergo more change relative to their pre-introduction configuration in the field.

## Methods

### Sample collection

**Fig. 1:**
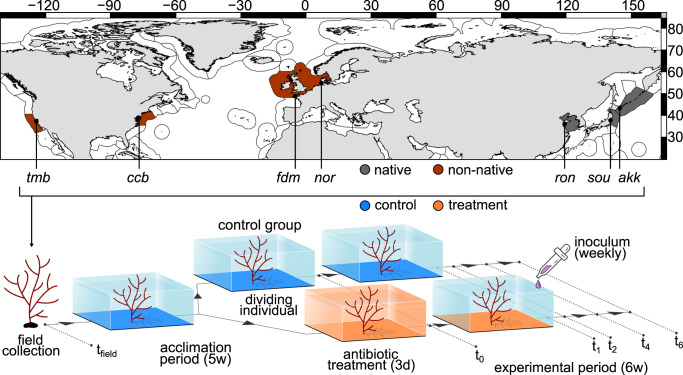
Schematic overview of the sampling design and experimental process. Algae were collected from native populations Rongcheng (*ron*), Soukanzan (*sou)* and Akkeshi *(akk*) and non-native populations Tomales Bay (*tmb*), Cape Charles Beach (*ccb*), Pleudihen-sur-Rance (*fdm*) and Nordstrand (*nor*). In the climate room algae were acclimated for 5 weeks and divided into two thalli. One of the thalli was treated for three days with an antibiotic mixture after which both groups were monitored for six weeks, during which the treated algae received inoculum with each water change. Microbiota samples were taken in the field (t_field_), directly after disturbance (t_0_) and after 1, 2, 4 and 6 weeks (t_1_, t_2_, t_4_ and t_6_).

Algae were sampled from August 27^th^ to September 21^st^ (2017) from seven populations also collected for Bonthond et al. [28], including three native populations; Akkeshi (Japan), Soukanzan (Japan), Rongcheng (China); and four non-native populations; Pleudihen-sur-Rance (France), Nordstrand (Germany), Cape Charles Beach (Viriginia) and Tomales Bay (California, Fig. 1, Table S1). Individuals fixed to hard substratum (see [30]) were sampled at least a meter apart from one another and stored in separate plastic bags. As *A. vermiculophyllum* has a complex, haplodiplontic life-cycle only diploids were included in the experiment. Life-cycle stages were identified in the field with a dissecting microscope or *post-hoc* by microsatellite genotyping [31]. After transport in coolers and storage at 4 °C in the lab, bags with algae were shipped to Germany, arriving within 4–6 days after collection. In the climate room (15 °C), individuals were transferred to separate transparent aquaria with transparent lids, containing 1.75 L artificial seawater (ASW) prepared from tap water and 24 gL^−1^ artificial sea salt without CaCO_3_ (high CaCO_3_ concentrations increase disease risk, Weinberger data unpublished) and exposed to 12 h of light per day (86.0 µmol m^−2^s^−1^ at the water surface). Aquaria were moderately aerated with aeration stones. Per population, four diploid individuals were acclimated over 31–32 days to climate room conditions prior to starting the experiment. Water was exchanged weekly with new ASW enriched with 2 mL Provasoli-Enrichment Solution (PES; [32]). At the start of the experiment, wet weight was recorded and individuals were divided into two parts of ~10 g each and placed into two plastic tanks with 1.75 L water and 2 mL PES (Fig. 1).

### Experimental setup

To rigorously disturb the microbial community, one of each of the pairs of aquaria containing the same algal individual was treated with a combination of antibiotics, aiming to increase the effectivity (10 mgL^−1^ ampicillin, 10 mgL^−1^ streptomycin, 10 mgL^−1^ chloramphenicol) and the other (control) remained untreated. All experimental work was conducted with disposable gloves and sterilized equipment, to minimize contamination. After three days, the water was removed from all tanks (treated and control) and the wet weight was recorded for all algae. All individuals were rinsed with one 1.75 L volume ASW and re-incubated in 1.75 L ASW. Subsequently, both groups received new ASW with 2 mL PES weekly and individuals treated with antibiotics received also 2 mL inoculum. The inoculum was prepared from individuals of all 7 populations, following the procedure to remove epibiota as described in Bonthond et al. [28]. Briefly, apical fragments of 1 g were separated from the thallus and transferred to 50 mL tubes containing 15 ± 1 glass beads (3 mm) and 15 mL ASW and vortexed for 6 min to separate epibiota from the algal tissue. In total, 8 samples were prepared from one individual per population. The resulting suspensions were pooled and mixed with glycerol (20% final glycerol concentration), aliquoted in 50 mL tubes and stored at −20 °C. For each water exchange, a new aliquot was defrosted at room temperature and added to the water of treated algae. Wet weight was recorded weekly with water exchanges. Before weighing the individual on aluminum foil, it was dipped twice on a separate aluminum foil sheet, to reduce attached water in a systematic way. Endo- and epiphytic microbiota were sampled in the field (t_field_, [28]), at the start of the experiment (t_0_), after one week (t_1_), two weeks (t_2_), four weeks (t_4_) and six weeks (t_6,_ Fig. 1). To equalize acclimation times across populations the experiment was stacked into five groups (Table S2). At each sampling moment, 0.5 or 1 g of tissue was separated from all individuals with sterilized forceps and epibiota were extracted similarly to the preparation of the inoculum. The resulting suspension was filtered through 0.2 µm pore size PCTA filters. Both the filters and the remaining tissue were preserved at −20 °C.

### DNA extraction and amplicon sequencing

Tissue samples were defrosted, rinsed with absolute ethanol and DNA free water to remove hydro- and moderately lipophilic cells and molecules from the surface and cut to fragments with sterilized scissors. DNA was then extracted from these fragments (endobiota) and from preserved filters (epibiota) using the ZYMO Fecal/soil microbe kit (D6102; ZYMO-Research, Irvine, CA, USA), following the manufacturer’s protocol. Although this method to separate endo- and epibiota was shown to resolve distinct communities [28], tightly attached epiphytic cells may not be completely removed from the surface and detectable in endophytic samples as well. Two 16S-V4 amplicon libraries, over which the samples were divided in a balanced manner, were prepared as in Bonthond et al. [28], following the two-step PCR strategy from Gohl et al. [33], using the same set of 16S-V4 target primers and indexing primers. The libraries were sequenced on the Illumina MiSeq platform (2×300 PE) at the Max-Planck-Institute for Evolutionary Biology (Plön, Germany), including four negative DNA extraction controls and four negative and positive PCR controls (mock communities; ZYMO-D6311). The fastq files were de-multiplexed (0 mismatches). Relevant field samples from Bonthond et al. [28] were combined with the new dataset and assembled, quality filtered and classified altogether with Mothur v1.43.0 [34] using the SILVA-alignment release 132 [35]. Sequences were clustered within 3% dissimilarity into OTUs using the opticlust algorithm. Mitochondrial, chloroplast, eukaryotic and unclassified sequences were removed. To prepare the community matrix we discarded singleton OTUs (in the full dataset), samples with <1000 read counts and OTUs from which all sequences were removed after the previous step. De-multiplexed reads and corresponding metadata were deposited in the SRA database (accession: PRJNA612003).

### Functional profiling

To predict functional groups we used Picrust2 software [36] with default settings. Using KO-numbers from the Kyoto encyclopedia of Genes and Genomes KEGG [37], we defined the following functional groups: autotrophy (*RuBisCo*; K01601), aerobic heterotrophy (COXIII; K02276), anaerobic heterotrophy (adenylyl-sulfate reductase; K00394, methane/ammonia monooxygenase; K10944 and fumarate reductase; K00244 combined) and diazotrophy (*nifH*; K02588).

### Identification of core microbiota

Geography-independent OTUs identified in Bonthond et al. [28] were re-identified in the new dataset by cross-comparing all OTU sequences with those of the core microbiota from the previous study [28]. Sequences that were identical, or most similar, were reclassified to epiphytic, endophytic or algal core OTUs.

### Statistical modeling

We used the relative growth rate (RGR) as a measure of performance. As tissue was removed from all individuals at each timepoint (t_0_–t_6_) RGR was obtained by dividing the gained wet weight by the weight after sampling at the previous timepoint and divided by the number of days in between sampling points (thus expressed in % growth d^−1^). RGR was analyzed with a linear mixed model as a function of *range*, *treatment*, *time* (weeks) and all interaction terms. To model the relationship between RGR and *time* in a flexible way *time* was included as a third order polynomial, therewith considering temporal variation, without imposing shapes more complex than a third order polynomial. We included *population*-*identity* and *individual-identity* as random intercepts to represent the genetic population structure by *A. vermiculophyllum* at the local scale [24] and to account for non-independence within individuals.

For alpha-diversity, we used OTU-richness rarefied to 1000 reads per sample and the probability of interspecific encounter (PIE) as a measure of evenness, obtained with the package *mobr* [38]. To compare diversity in the field with the beginning of the experiment, field and control samples from the first timepoint were used, including *substrate* and *time* as fixed and *population* and *individual-identity* as random effects. Then, we fitted third order polynomial functions of *time* on the subset of the data including experiment samples (t_0–6_). These models also included the predictors *substrate*, *treatment* and random intercepts *population-* and *individual-identity*. To meet normality, PIE was logit transformed. To account for possible effects resulting from differences in read counts across samples, the log of the sequencing depth (LSD) was included in all models as a continuous variable. Predicted functional groups were analyzed with the same model structures. To meet normality, responses were log (+1 when including zeros) or squared-root transformed.

To analyze community composition between treatments we used multivariate generalized linear models (mGLMs) from the R package mvabund [39] in a two-step approach. The community matrices were trimmed to the 95% most abundant OTUs and split by substrate to analyze epi- and endobiota separately. First, a mGLM was used to remove the effects of sequencing depth (by including LSD) and differences among populations (by including *population-identity*). The mGLMs assumed a negative binomial distribution with a log in the link function. Second, on the residuals we ran a mGLM in response to *treatment*, *time* (a third order polynomial) and the interaction. This model assumed a Gaussian distribution as the residuals from the first model were normally distributed in the link function. Multivariate statistics were obtained by resampling the univariate models with 500 bootstrap iterations. Compositional differences between control and treatment over time were visualized using non-metric multidimensional scaling (nMDS) on the rescaled residuals of the first model. Group centroids and corresponding 95% confidence regions were computed with the R package vegan [40]. Compositional changes at the univariate level (i.e., specific OTUs) were visualized with a heatmap including the fifty most abundant OTUs by treatment and timepoint.

Beta-diversity was analyzed with pairwise Bray–Curtis distances from the epi- and endophytic datasets that were adjusted for the sequencing depth using mGLMs as a function of LSD. Additionally, we ran these models on weighted UniFrac distances, for which representative sequences of all OTUs were aligned with MAFFT v7.221 [41], with *Saccharomyces cerevisiae* as outgroup, and clustered into a maximum-likelihood tree with RAxML v8.2.12 [42] with the GTR + G substitution model and a 1000 bootstrap iterations. We compared distances among individuals, calculated between samples within the same timepoint (i.e., t_0_–t_0_, t_1_–t_1_, etc.) and regressed those against a third order polynomial of *time*, a new factor *population* (levels: *within-* and *between-populations*), *range* (*native* and *non-native*), *treatment* (*control* and *treated*) and all interactions. The random intercepts *population-combination* and *individual-combination* were included to account for non-independence resulting from calculating distances by making different combinations with the same individuals. To characterize how community composition changed with respect to the composition observed in the field, we calculated Bray–Curtis and weighted UniFrac distances between individuals in the experiment (t_0_–t_6_) and individuals in the field (t_field_) within the same population. These models (for endo- and epibiota separately) included a third order polynomial of *time*, the *range*, their interaction and the random intercept *individual-combination*.

Proportional changes in core OTU abundances were analyzed with a mixed linear model using the subset of our data containing only field samples and the final timepoints (t_6_) and included the variables *treatment*, *substrate, range* and the interactions. *Population-* and *individual-identity* were included as random intercepts. All univariate analyses were conducted using the R package lme4 [43], calculating marginal and conditional R^2^ values (variation explained by fixed effects and fixed plus random effects, respectively) with the r.squaredGLMM function [44]. Violations of model assumptions were verified visually with *QQ-plots* and *residual-vs-fitted-plots* for univariate and multivariate analyses.

## Results

The final OTU matrix counted 14,287 OTUs and 4,688,853 reads. The sequencing depth ranged from 1005 to 77,922 (median = 6702). Proteobacteria was the most abundant phylum, followed by Bacteroidetes, Planctomycetes and Cyanobacteria. Whereas Bacteroidetes was most abundant in the treated algae, controls were dominated by Proteobacteria. Also Cyanobacteria were more pronounced in the controls, especially in endobiota (Fig. S1). We successfully re-identified 185 core OTUs from Bonthond et al. [28] in the new dataset (Table S3). The overall most abundant OTU (classified to *Alteromonas*) was prevalent both in controls and treated algae during the first weeks of the experiment, but declined again during the final weeks. By the end of the experiment epi- and endophytic communities of treated algae were dominated by OTU3 (*Maribacter*), which was of low abundance in the field, and OTU4 (also *Maribacter*), which was also prevalent in field samples. Core microbiota dominant in field samples, including OTU2 (*Granulosicoccus*), OTU8 (*Erythrobacter*) and OTU10 (*Pleurocapsa*) remained abundant in controls but declined in treated algae.

The proportional abundance of core OTUs differed between substrates and treatments (*p* < 0.001) but not between ranges (*p* = 0.633; Table S4A). Core OTUs constituted a mean proportion of 0.577 (95% confidence interval: 0.539–0.615) in endobiota, compared to 0.386 (0.358–0.414) in epibiota. The proportional abundance of core OTUs decreased during the experiment in controls and treated algae from 0.629 (0.595–0.662) to 0.285 (0.243–0.328) in the controls and 0.340 (0.297–0.383) in treated algae. The difference between controls and treated algae was not significant (*p* = 0.061).

### Host performance

**Fig. 2:**
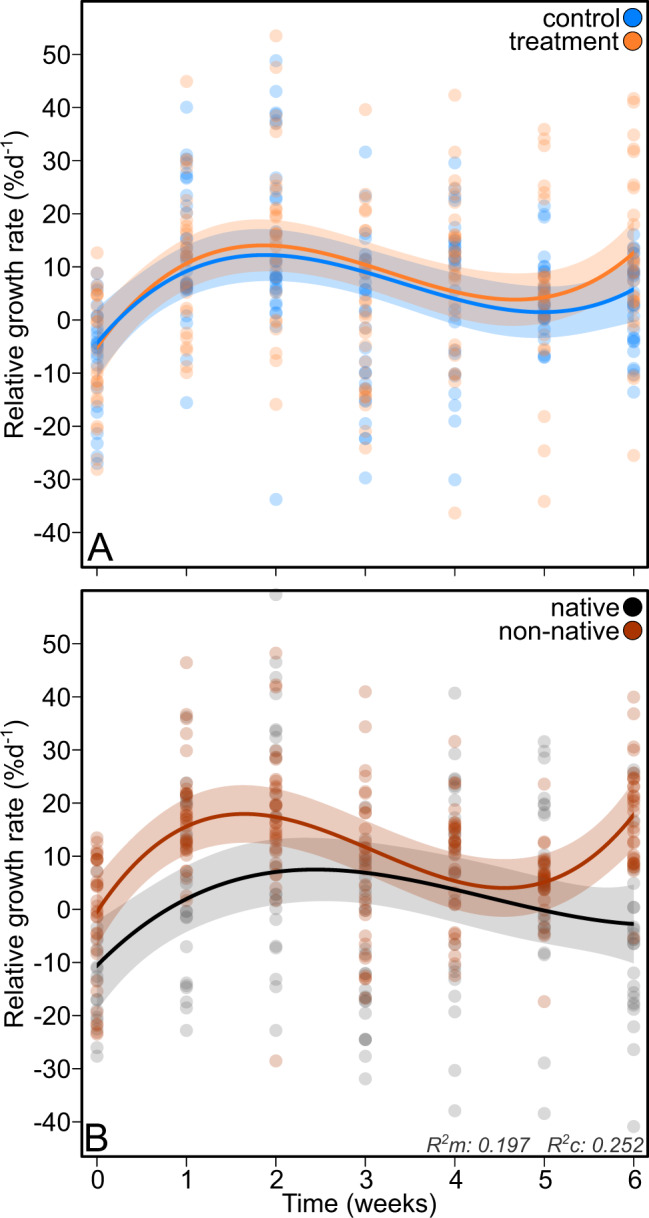
Regression curves of the relative growth rate (RGR; in %day^−1^). Trends are shown by treatment (**A**) and by range (**B**) over the duration of the experiment, displaying control (blue) and treatment groups (orange) and native (gray) and non-native algae (red). The 95% confidence regions are indicated in shades with the corresponding color. Marginal and conditional R^2^ values of the model are displayed in the bottom right corner of panel **B**.

RGR varied with time (*p* < 0.001; Table S4B), increasing steadily over the first 1-2 weeks, decreasing somewhat over the 2-3 subsequent weeks and increasing again during the last weeks. Controls and treated algae did not differ in RGR (*p* = 0.162; Fig. 2A), but RGR was higher in non-native hosts (*p* = 0.043) and varied between ranges with time (*p* = 0.002; Fig. 2B). During the first weeks RGR increased for both groups, although substantially faster for non-natives. Following this increase RGR dropped and became even negative among native algae while non-native hosts recovered and reached again high RGRs.

### Alpha-diversity and predicted functional groups

**Fig. 3:**
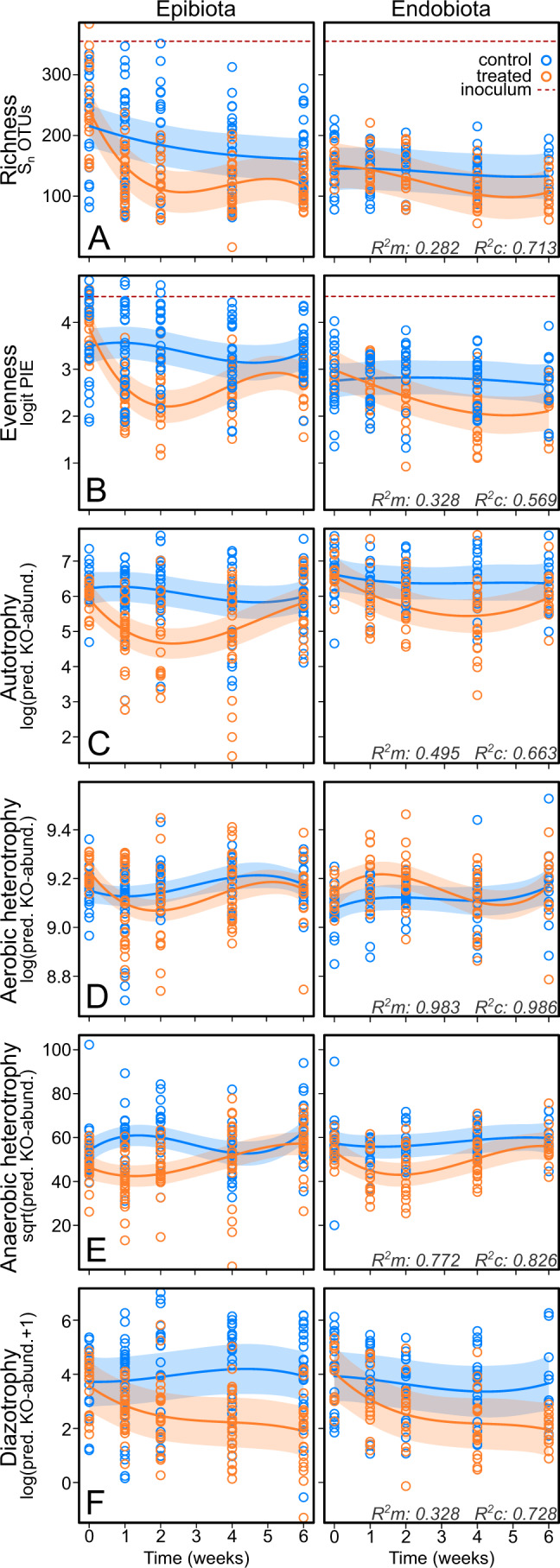
Regression curves of alpha-diversity and predicted functional group abundances. Plots are separated by substrate (epibiota on the left and endobiota on the right) and the controls (blue) and treated algae (orange) are shown in the same panel. For alpha-diversity, rarefied richness (S_n_) and PIE as a measure of evenness were analyzed (**A**, **B**). The rarefied richness and PIE values of the inoculum are displayed inside the same figure by the red dashed line. The predicted functional groups are derived from predicted abundance of functional KEGG orthologs (KOs) and include autotrophy (**C**), aerobic heterotrophy (**D**), anaerobic heterotrophy (**E**) and diazotrophy (**F**). 95% confidence regions are drawn in shades with a color corresponding to the treatment and marginal and conditional R^2^ values are shown in the bottom right corners.

In the course of the experiment, epibiotic OTU-richness decreased slowly in the controls, but more steeply in the treated samples, recovering slightly towards the end of the experiment. OTU-richness in endophytic communities remained stable in the controls, but decreased in the treated samples (Fig. 3A). Consequently, the difference in richness between epi- and endobiota observed in the field ([28], Fig. S2) also decreased and rarefied richness in both substrates was similar when the experiment ended. There was an overall effect of substrate (*p* < 0.001, Table S4C). Also in terms of evenness, controls remained stable while treated algae decreased (Fig. 3B). Generally, evenness was lower in the tissue than the surface (*p* < 0.001). LSD was not significant for either rarefied richness or evenness and was therefore excluded from the models.

In the process of predicting functional profiles, 1908 OTUs had NSTI values above 2 and were, together with one poorly aligned OTU, excluded from downstream analyses. Predicted autotrophy, anaerobic heterotrophy and diazotrophy were overall lower in treated epi- and endobiota and behaved differently with time (*p* < 0.001, Table S4D, Fig. 3C–F). Autotrophy and anaerobic heterotrophy decreased initially in treated algae but eventually recovered to levels equal to controls. Diazotrophy decreased in the treatment group and remained below that of the control over the duration of the experiment, especially in epibiota. Overall, there was no difference between control and treated algae in predicted aerobic heterotrophy. However, the interaction of treatment with time was significant (*p* = 0.035, Fig. 3C–F, Table S4D).

### Community composition

**Fig. 4:**
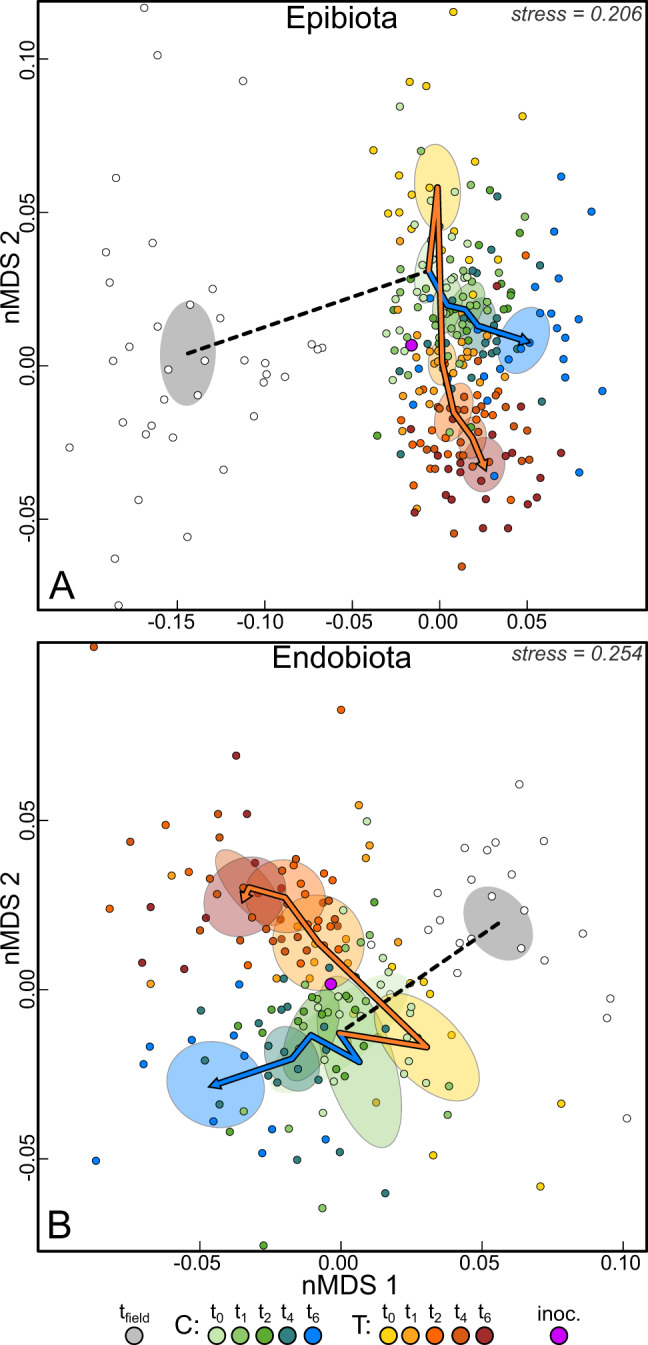
nMDS plots of community composition based on the rescaled residuals of mGLMs removing the effects of *LSD* and *population-identity*. Epibiota (**A**) and endobiota (**B**) were analyzed separately. Note that two outlier datapoints in **B** are not within the limits of the frame. 95% confidence regions of the centroids in the two-dimensional space of all data points from the same treatment (abbreviated in the legend with C and T) and timepoint (t_field_ – t_6_) are shown in shaded ellipses. In both diagrams the inoculum (purple) was included in the scaling. The centroid of all field samples is connected by a dashed line to the centroid of the control group at t_0_, representing the composition before the treatment. From the control group at t_0_, trajectories are drawn through the centroids of controls (green-blue) and treated algae (yellow-red) to display the changes in community composition over time. Stress values are displayed in the upper right corners.

Both endo- and epibiota of treated algae differed compositionally from the controls (*p*_epi_ = 0.004, *p*_endo_ = 0.004) and also responded differently over time (*p* = 0.004; Table S4E). This was also observed in the nMDS plots, where the centroids of the controls and treated algae clustered apart and had diverging trajectories (Fig. 4). A few taxa became highly abundant in the treatment group (Fig. S1). These highly abundant OTUs were classified to *Alteromonas* (OTU1; Gammaproteobacteria) and *Maribacter* (OTU3 and OTU4, Bacteroidetes), of which OTU3 was dominant at the end of the experiment in both epi- and endobiota. OTU1 was also prevalent in controls, but those were dominated by OTU8 (a core OTU classified to *Erythrobacter*), which were of low abundance in the treated algae. Additionally, the core-members OTU5 (*Paraglaciecola*) and OTU9 (Rhodobacteraceae) were abundant in controls. The endophytic core OTUs 2 and 10 (*Granulosicoccus* and *Pleurocapsa*, respectively), were prevalent in the samples from the field and remained of major abundance in controls but declined in treated algae.

### Beta-diversity

**Fig. 5:**
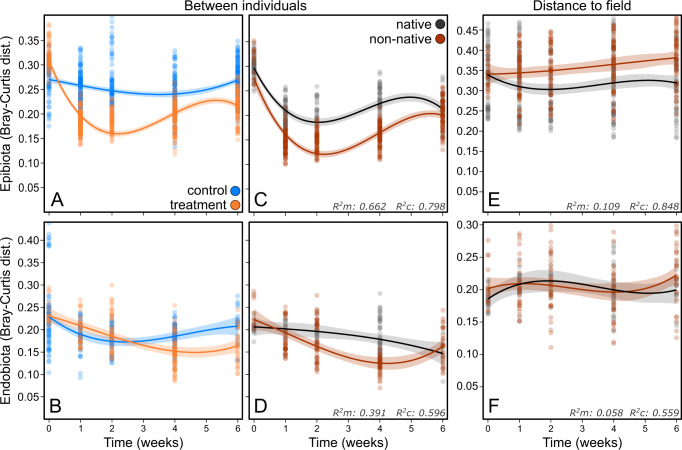
Beta-diversity regression curves. Bray–Curtis distances in microbial community composition over the course of the experiment for epi- (**A**–**C**; from the same model) and endobiota (**B**–**D**; from the same model). Diagrams display the mean distance between populations over time, within controls (blue) and within treated algae (orange; panels **A** and **B**) between treated native (black) and treated non-native (red) individuals over time (**C** and **D**) and the distance between community compositions in the experiment and the field (**E** and **F**). The 95% confidence regions are indicated in shades of the corresponding color. Marginal and conditional R^2^ values of the models are displayed in the bottom right of the most right panel originating from the same model.

Epiphytic beta-diversity among populations – measured in Bray–Curtis distances between individuals from different populations – remained approximately constant in the controls, but declined steeply in the treated algae immediately after the treatment. During the last two weeks of the experiment beta-diversity among populations recovered somewhat in the treated algae, but not to the level of the controls (Fig. 5A). Epiphytic beta-diversity within populations was overall lower than between populations (*p* < 0.001), but followed a similar trend with a stable mean distance in the controls and a decrease in mean distance among treated algae (Fig. S3A). Trends and statistical output from models ran on Bray–Curtis distances were generally similar to those obtained from weighted UniFrac distances (see Fig. S3 and Table S4F for details). Within treated algae, epiphytic beta-diversity was substantially lower among non-native populations (*p* < 0.001). In native and non-native populations, distances decreased during the first weeks post-disturbance. However, the decline was more rapid and reached overall lower levels among non-natives. Bray–Curtis distances increased again in both groups after two weeks, but remained lower among non-native holobionts (Fig. 5C, Table S4F). Overall, weighted UniFrac distances were lower in treated non-native holobionts as well but became similar to natives during the final week of the experiment (Fig. S3O, Table S4F).

Trends in endophytic beta-diversity were similar, but less pronounced. The mean distance between populations decreased weakly in both controls and treated algae during the first two weeks, after which the controls began to recover. In the treated algae the mean distance remained low, resulting in an overall difference between controls and treated algae (*p* = 0.003, Fig. 5B). Endophytic beta-diversity was overall lower in non-native individuals (*p* = 0.007, Fig. 5D).

Compared to communities in the field, epibiota of non-native individuals were more dissimilar than native individuals (*p* = 0.001). This dissimilarity increased during the experiment, whereas it remained approximately stable among native individuals (Fig. 5E), resulting in a significant interaction between time and range (*p* < 0.001). Bray–Curtis distance relative to the field for endobiota did not differ between native and non-native holobionts (*p* = 0.561). However, the interaction was marginally significant (*p* = 0.04996), possibly reflecting an increase in mean distance in non-native holobionts during the last timepoint (Fig. 5F, Table S3G, Fig. S4).

## Discussion

### The generalist host hypothesis

Non-native hosts performed better than native hosts (Fig. 2), which supports the first sub-hypothesis outlined in the introduction and demonstrates that non-native populations are more capable of enduring holobiont disturbance and introduction to a new environment. Furthermore, our data also support the second sub-hypothesis that post-disturbance beta-diversity was lower among non-native populations. Microbial communities followed converging succession trajectories in the common garden, leading to a *common* community specific to the environment. Communities of non-native holobionts converged more and thus configured *more* *common* epi- and endobiota. The third sub-hypothesis – that microbiota of native holobionts remain more similar to the community observed in the field – was supported for epiphytic communities, suggesting that native hosts are indeed less flexible and depend more on the pre-invasion community. For endobiota this sub-hypothesis was rejected, as changes in microbiota were not detectably different between native and non-native holobionts. Therefore, our results demonstrate an increase in *host flexibility* in non-native populations, specifically towards epibiota. This is in line with the prediction of the generalist host hypothesis that increased host promiscuity promotes invasiveness and provides experimental evidence this may be an important trait in seaweed invasions.

### Holobiont disturbance

The applied disturbance had a strong impact, resulting in different microbial communities. While, with the exception of aerobic heterotrophs, communities of treated algae shifted functionally at the beginning, recovery of autotrophy and anaerobic heterotrophy toward levels in the control group by the end of the experiment suggests these differentiated communities were to a substantial degree functionally redundant. Hence, controls and treated algae performed equally well (Fig. 2A). Some OTUs thrived in treated holobionts. In particular, two *Maribacter* OTUs became dominant and also a number of OTUs classified to Rhodobacteraceae increased in prevalence. In green algae of the genus *Ulva*, morphogenesis has been shown to partially depend on a tripartite interaction with bacteria from *Maribacter* and *Roseovarius* (Rhodobacteracea) which are acquired from the environment [45]. While we can only speculate, the strong post-disturbance shifts observed in *Maribacter* and Rhodobacteraceae OTUs might hint that also in Rhodophyta these environmentally widespread bacteria may be functionally relevant to the holobiont. Rhodobacteraceae –prevalent in the *A. vermiculophyllum* core as well ([28], Fig. S1)– are commonly associated with macro-algae [46] and metabolize algal osmolytes such as dimethylsulfoniopropionate [47]. Whereas some Rhodobacteraceae OTUs decreased in treated algae, others thrived. Such shifts of closely related taxa may reflect functionally redundant substitutions inside the holobiont, for which the host requires some degree of promiscuity, as implied by the generalist host hypothesis. Moreover, given the metabolic diversity of this group [46], some of the Rhodobacteraceae OTUs that prospered in the treatment might even be able to substitute more distant taxa such as for instance core OTU2, classified to *Granulosicoccus*, a genus also equipped to metabolize dimethylsulfoniopropionate [48].

While succession in treated holobionts resulted in communities that were in auto- and heterotrophic respects similar to controls, diazotrophy did not recover to control group levels. Interestingly, this may either indicate that diazotophy in the community is simply irrelevant to the holobiont, or that the host is not as promiscuous to nitrogen-fixing members as it is to other functional groups. However, experimental work with specific isolates is needed to identify to which microbial groups flexibility in non-native holobionts may have increased and which mechanisms underlay host promiscuity. Given that members of the *Pleurocapsa*-group synthesize nitrogenase [49], these abundant core members are then of specific interest.

### Epi- and endobiota

Overall, trends were less pronounced in endobiota, which may indicate this micro-environment was less affected by the disturbance or may harbor a more resistant community. While significant, the difference in beta-diversity between native and non-native populations was also not as strongly pronounced in endobiota as it was in epibiota. Furthermore, we observed that geographically conserved symbionts (core microbiota) are proportionally more abundant in the tissue than on the surface and their abundance did not differ between disturbed and undisturbed holobionts. Endobiota are likely less exposed to environmental and microbial pressures in general and to disturbances such as those during transport [17] or as the one applied here. It is conceivable that while flexibility may have increased towards epibiota, the more protected tissue of *A. vermiculophyllum* may harbor microbes toward which the host is less promiscuous and of which the co-introduction may be more important for a successful invasion. Arnaud-Haond et al. [50] showed that for the green alga *Caulerpa taxifolia* specifically endobiotia had accompanied their host in the invasion process. Especially for seaweeds, which live in continuous exposure to high numbers of microbes trying to colonize their tissue [7], putatively important microbial mutualists may be more protected endophytically and therewith less likely lost upon disturbance. Therefore, while our results show increased host flexibility in non-native *A. vermiculophyllum* populations towards epibiota, they may at the same time hint at *accompanied mutualists* residing endophytically.

### Host traits

This study is to our knowledge the first to provide experimental evidence that host promiscuity may be an important trait in macroalgal invasions, as it is for terrestrial plants [20, 21]. It therewith supports the applicability of the generalist host hypothesis in a marine species. However, it remains unclear which host-mechanisms underlie promiscuous phenotypes. Future studies are needed to test whether the observed intraspecific increase in host promiscuity is linked to mechanisms of defense against epiphytic settlers [25–27], to the production of metabolites attracting specific taxa [11], or to other mechanisms. Ultimately, addressing whether differences in these traits between the ranges are adaptive and/or whether they are more plastic among non-native populations may identify processes of general relevance to invasive holobionts.

Increased growth rate is a trait typically associated with biological invasions [51]. The shape of the trends in the timeseries of the present study is noteworthy and may suggest that initial growth rates are supported by the use of energy reserves, which are depleted in the course of the experiment. Then, where growth continues to drop for native holobionts, non-native holobionts recover their growth, possibly reflecting a difference between native and non-native holobionts in their ability to successfully establish.

## Conclusions

In the present work, an experimentally simulated invasion demonstrated that microbial succession trajectories of different *A. vermiculophyllum* populations converge toward a new microbial community specific to the new environment. Non-native *A. vermiculophyllum* populations were more *promiscuous* toward epibiota and showed superior immediate and long-term performance. This intraspecific increase in host promiscuity is supported by two lines of evidence. First, non-native holobionts configure more common epiphytic microbial communities in the common garden compared to native holobionts. In other words, upon introduction, they reconfigure a community more effectively. Second, epibiota of native populations remain more similar to their natural composition, supporting that native holobionts are more dependent on the configuration in the original environment and less flexible toward change. Therefore, our results demonstrate that, similar to plants, host promiscuity can be an important trait among invasive macroalgae. We posit in conclusion that the *generalist host hypothesis* may be commonly applicable in algal invasions and warrants further investigation.

## Supplementary information

Supplementary materials

## Data Availability

The raw de-multiplexed V4-16S gene amplicon reads and associated metadata are available from the SRA database under the Bioproject accession number PRJNA612003.

## References

[1] Vitousek PM, Walker LR, Whiteaker LD, Mueller-Dombois D, Matson PA (1987). Biological invasion by *Myrica faya* alters ecosystem development in Hawaii. Science..

[2] Pimentel D, Zuniga R, Morrison D (2005). Update on the environmental and economic costs associated with alien-invasive species in the United States. Ecol Econ..

[3] Simberloff D, Martin J, Genovesi P, Maris V, Wardle DA, Aronson J (2013). Impacts of biological invasions: what’s what and the way forward. Trends Ecol Evol.

[4] van der Putten, Wim H, Klironomos JN, Wardle DA (2007). Microbial ecology of biological invasions. ISME J.

[5] Grosholz E (2002). Ecological and evolutionary consequences of coastal invasions. Trends Ecol Evol.

[6] Williams SL, Smith JE (2007). A global review of the distribution, taxonomy, and impacts of introduced seaweeds. Annu Rev Ecol Evol Syst..

[7] Wahl M, Goecke F, Labes A, Dobretsov S, Weinberger F (2012). The second skin: ecological role of epibiotic biofilms on marine organisms. Front Microbiol.

[8] Bordenstein SR, Theis KR (2015). Host biology in light of the microbiome: ten principles of holobionts and hologenomes. PLoS Biol.

[9] Badri DV, Vivanco JM (2009). Regulation and function of root exudates. Plant Cell Environ.

[10] Longford SR, Campbell AH, Nielsen S, Case RJ, Kjelleberg S, Steinberg PD (2019). Interactions within the microbiome alter microbial interactions with host chemical defences and affect disease in a marine holobiont. Sci Rep.

[11] Saha M, Weinberger F (2019). Microbial “gardening” by a seaweed holobiont: surface metabolites attract protective and deter pathogenic epibacterial settlement. J Ecol.

[12] Harder T, Campbell AH, Egan S, Steinberg PD (2012). Chemical mediation of ternary interactions between marine holobionts and their environment as exemplified by the red alga *Delisea pulchra*. J Chem Ecol.

[13] Pietschke C, Treitz C, Foret S, Schultze A, Kunzel S, Tholey A (2017). Host modification of a bacterial quorum-sensing signal induces a phenotypic switch in bacterial symbionts. Proc Natl Acad Sci USA..

[14] Nguyen C (2003). Rhizodeposition of organic C by plants: mechanisms and controls. Agronomie..

[15] Rosier A, Bishnoi U, Lakshmanan V, Sherrier DJ, Bais HP (2016). A perspective on inter-kingdom signaling in plant-beneficial microbe interactions. Plant Mol Biol.

[16] Nuñez MA, Horton TR, Simberloff D (2009). Lack of belowground mutualisms hinders *Pinaceae* invasions. Ecology..

[17] Bax N, Williamson A, Aguero M, Gonzalez E, Geeves W (2003). Marine invasive alien species: a threat to global biodiversity. Mar Policy.

[18] Blackburn TM, Pyšek P, Bacher S, Carlton JT, Duncan RP, Jarošík V (2011). A proposed unified framework for biological invasions. Trends Ecol Evol.

[19] Rodríguez‐Echeverría S (2010). Rhizobial hitchhikers from Down Under: invasional meltdown in a plant–bacteria mutualism?. J Biogeogr..

[20] Rodríguez-Echeverría S, Le Roux JJ, Crisóstomo JA, Ndlovu J (2011). Jack‐of‐all‐trades and master of many? How does associated rhizobial diversity influence the colonization success of Australian *Acacia* species?. Divers Distrib..

[21] Klock MM, Barrett LG, Thrall PH, Harms KE (2015). Host promiscuity in symbiont associations can influence exotic legume establishment and colonization of novel ranges. Divers Distrib..

[22] Parker MA (2001). Mutualism as a constraint on invasion success for legumes and rhizobia. Divers Distrib..

[23] Perret X, Staehelin C, Broughton WJ (2000). Molecular basis of symbiotic promiscuity. Microbiol Mol Biol Rev..

[24] Krueger-Hadfield SA, Kollars NM, Strand AE, Byers JE, Shainker SJ, Terada R (2017). Genetic identification of source and likely vector of a widespread marine invader. Ecol Evol.

[25] Saha M, Wiese J, Weinberger F, Wahl M (2016). Rapid adaptation to controlling new microbial epibionts in the invaded range promotes invasiveness of an exotic seaweed. J Ecol.

[26] Wang S, Weinberger F, Xiao L, Nakaoka M, Wang G, Krueger-Hadfield SA (2017). In situ common garden assays demonstrate increased defense against natural fouling in non-native populations of the red seaweed *Gracilaria vermiculophylla*. Mar Biol.

[27] Wang S, Wang G, Weinberger F, Bian D, Nakaoka M, Lenz M (2017). Anti-epiphyte defences in the red seaweed *Gracilaria vermiculophylla*: non-native algae are better defended than their native conspecifics. J Ecol.

[28] Bonthond G, Bayer T, Krueger-Hadfield SA, Barboza FR, Nakaoka M, Valero M (2020). How do microbiota associated with an invasive seaweed vary across scales?. Mol Ecol..

[29] Zaneveld JR, McMinds R, Thurber RV (2017). Stress and stability: applying the Anna Karenina principle to animal microbiomes. Nat Microbiol.

[30] Krueger-Hadfield SA, Stephens TA, Ryan WH, Heiser S (2018). Everywhere you look, everywhere you go, there’s an estuary invaded by the red seaweed *Gracilaria vermiculophylla* (Ohmi) Papenfuss, 1967. BioInvasions Rec.

[31] Krueger-Hadfield SA, Kollars NM, Byers JE, Greig TW, Hammann M, Murray DC (2016). Invasion of novel habitats uncouples haplo-diplontic life cycles. Mol Ecol..

[32] Starr RC, Zeikus JA (1993). UTEX—The Culture collection of algae at the University of Texas at Austin 1993 list of cultures. J Phycol.

[33] Gohl DM, Vangay P, Garbe J, MacLean A, Hauge A, Becker A (2016). Systematic improvement of amplicon marker gene methods for increased accuracy in microbiome studies. Nat Biotechnol.

[34] Schloss PD, Westcott SL, Ryabin T, Hall JR, Hartmann M, Hollister EB (2009). Introducing mothur: open-source, platform-independent, community-supported software for describing and comparing microbial communities. Appl Environ Microbiol.

[35] Quast C, Pruesse E, Yilmaz P, Gerken J, Schweer T, Yarza P (2013). The SILVA ribosomal RNA gene database project: improved data processing and web-based tools. Nucleic Acids Res.

[36] Douglas GM, Maffei VJ, Zaneveld JR, Yurgel SN, Brown JR, Taylor CM, et al. PICRUSt2 for prediction of metagenome functions. Nat Biotechnol. 2020;38:685–8. 10.1038/s41587-020-0548-6. 10.1038/s41587-020-0548-6 PMC7365738 32483366

[37] Kanehisa M, Goto S, Sato Y, Kawashima M, Furumichi M, Tanabe M (2013). Data, information, knowledge and principle: back to metabolism in KEGG. Nucleic Acids Res.

[38] McGlinn DJ, Xiao X, May F, Gotelli NJ, Engel T, Blowes SA (2018). Measurement of Biodiversity (MoB): a method to separate the scale-dependent effects of species abundance distribution, density, and aggregation on diversity change. Methods Ecol Evol.

[39] Wang Y, Naumann U, Wright ST, Warton DI (2012). mvabund–an R package for model-based analysis of multivariate abundance data. Methods Ecol Evol.

[40] Oksanen J, Blanchet FG, Kindt R, Legendre P, Minchin PR, O’hara R, et al. Package ‘vegan’. Community Ecol package, version. 2013;2:9.

[41] Katoh Standley (2013). MAFFT multiple sequence alignment software version 7: improvements in performance and usability. Mol Biol Evolution.

[42] Stamatakis A. RAxML Version 8: a tool for phylogenetic analysis and post-analysis of large phylogenies. Bioinformatics. 2014. 10.1093/bioinformatics/btu033. 10.1093/bioinformatics/btu033 PMC3998144 24451623

[43] Bates D, Mächler M, Bolker B, Walker S (2015). Fitting linear mixed-effects models using lme4. J Stat Softw.

[44] Nakagawa S, Schielzeth H (2013). A general and simple method for obtaining R2 from generalized linear mixed-effects models. Methods Ecol Evol.

[45] Alsufyani T, Califano G, Deicke M, Grueneberg J, Weiss A, Engelen AH (2020). Macroalgal–bacterial interactions: identification and role of thallusin in morphogenesis of the seaweed *Ulva* (Chlorophyta). J Exp Bot..

[46] Dogs M, Wemheuer B, Wolter L, Bergen N, Daniel R, Simon M (2017). Rhodobacteraceae on the marine brown alga *Fucus spiralis* are abundant and show physiological adaptation to an epiphytic lifestyle. Syst Appl Microbiol.

[47] Moran MA, Reisch CR, Kiene RP, Whitman WB (2012). Genomic insights into bacterial DMSP transformations. Ann Rev Mar Sci.

[48] Kang I, Lim Y, Cho J (2018). Complete genome sequence of *Granulosicoccus antarcticus* type strain IMCC3135T, a marine gammaproteobacterium with a putative dimethylsulfoniopropionate demethylase gene. Mar Genom.

[49] Rippka R, Waterbury J, Herdman M, Castenholz R. *Pleurocapsa*- group. In: Bergey’s Manual of Systematics of Archaea and Bacteria, Wiley Online Library; 2015. pp 1–9. 10.1002/9781118960608.

[50] Arnaud-Haond S, Aires T, Candeias R, Teixeira S, Duarte CM, Valero M (2017). Entangled fates of holobiont genomes during invasion: nested bacterial and host diversities in *Caulerpa taxifolia*. Mol Ecol..

[51] Van Kleunen M, Weber E, Fischer M (2010). A meta‐analysis of trait differences between invasive and non‐invasive plant species. Ecol Lett..

