# Supplementary material for: The role of host promiscuity in the invasion process of a seaweed holobiont
Source: ISME J. 2021 Jan 21;15(6):1668–79. doi: 10.1038/s41396-020-00878-7 (PMC8163768; doi:10.1038/s41396-020-00878-7)
Supplement: Supplementary file 1 — Supplementary materials [file 41396_2020_878_MOESM1_ESM.pdf]

Supplemental Information:

The role of host promiscuity in the invasion process of a seaweed holobiont

Guido Bonthond[1]*, Till Bayer[1], Stacy A. Krueger-Hadfield[2], Nadja Stärck[1], Gaoge Wang[3], Masahiro Nakaoka[4], Sven Künzel[5], Florian Weinberger[1]

[1] GEOMAR Helmholtz Centre for Ocean Research Kiel, Düsternbrooker Weg 20, 24105, Kiel, Germany.

[2] Department of Biology, University of Alabama at Birmingham, 1300 University Blvd, CH464, Birmingham, AL, 35294, USA

[3] College of Marine Life Sciences and Institute of Evolution and Marine Biodiversity, Ocean University of China, 5 Yushan Road, Qingdao 266003, China

[4] Akkeshi Marine Station, Field Science Center for Northern Biosphere, Hokkaido University, Aikappu 1, Akkeshi, Hokkaido 088-1113, Japan

[5] Max Planck Institute for Evolutionary Biology, Plön, Germany

* Corresponding author: gbonthond@geomar.de

Table of Contents:

**Figure S1**	Page 2
**Figure S2**	Page 3
**Figure S3**	Page 4
**Figure S4**	Page 5
**Table S1** Sample summary	Page 6,7
**Table S2** Experiment overview	Page 8-10
**Table S3** Core OTUs	Page 11-15
**Table S4** Statistical output	Page 16-22

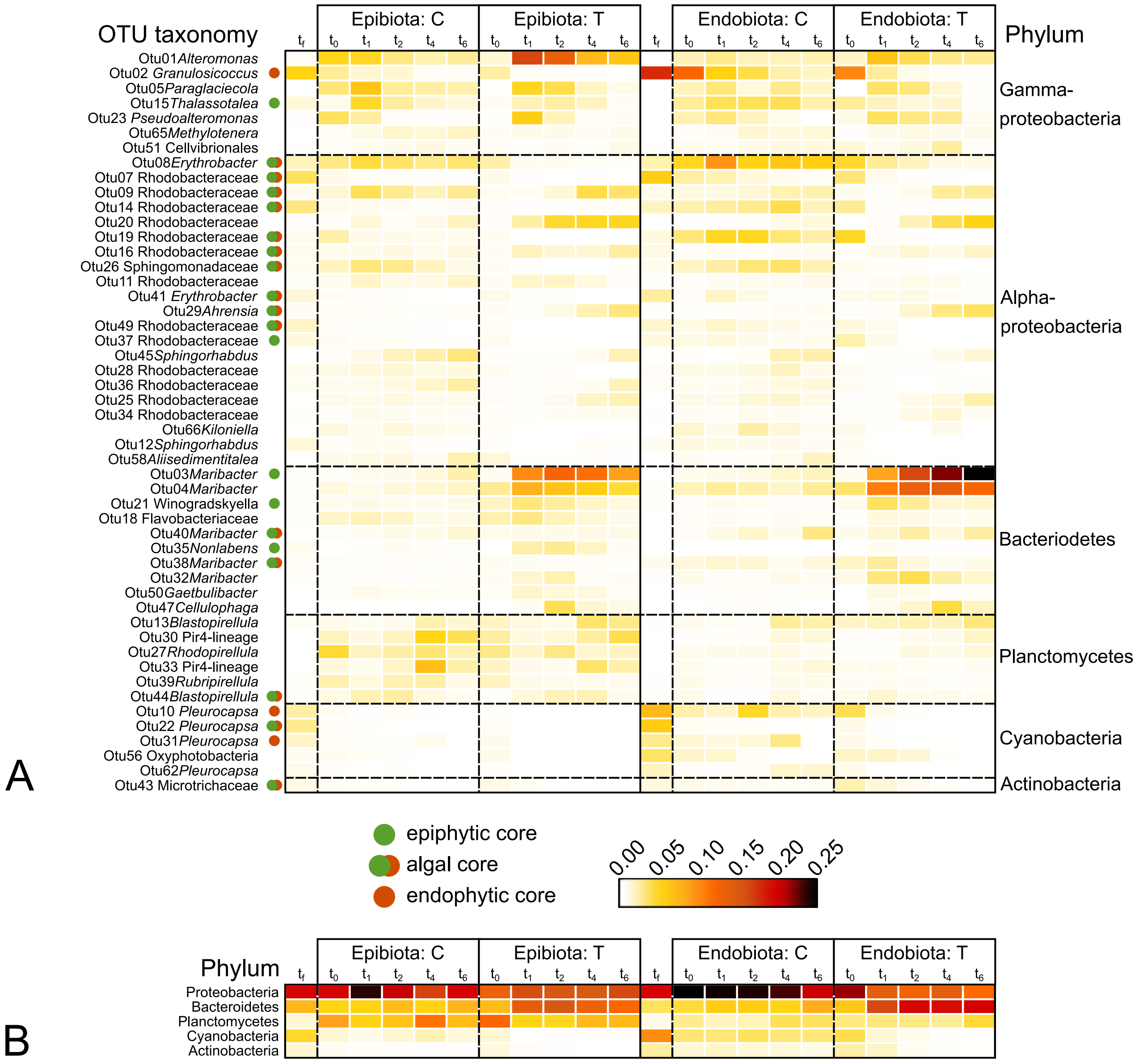

Figure S1. Relative OTU (A) and Phyla (B) abundances displayed in a heatmap by substrate (epi- and endobiota) and treatment (control and treated). Only the 50 most abundant OTUs and 5 most abundant Phyla are shown. Columns are ordered in time, starting from left right with the field collection (t_f) and ending with the final sampling in the experiment (t_6). Rows are ordered by Phylum and abundance. Core OTUs, identified as geographically independent in Bonthond et al. (2020), are labeled with green (epiphytic core), red or green-red (geographically conserved in both epi- and endobiota).

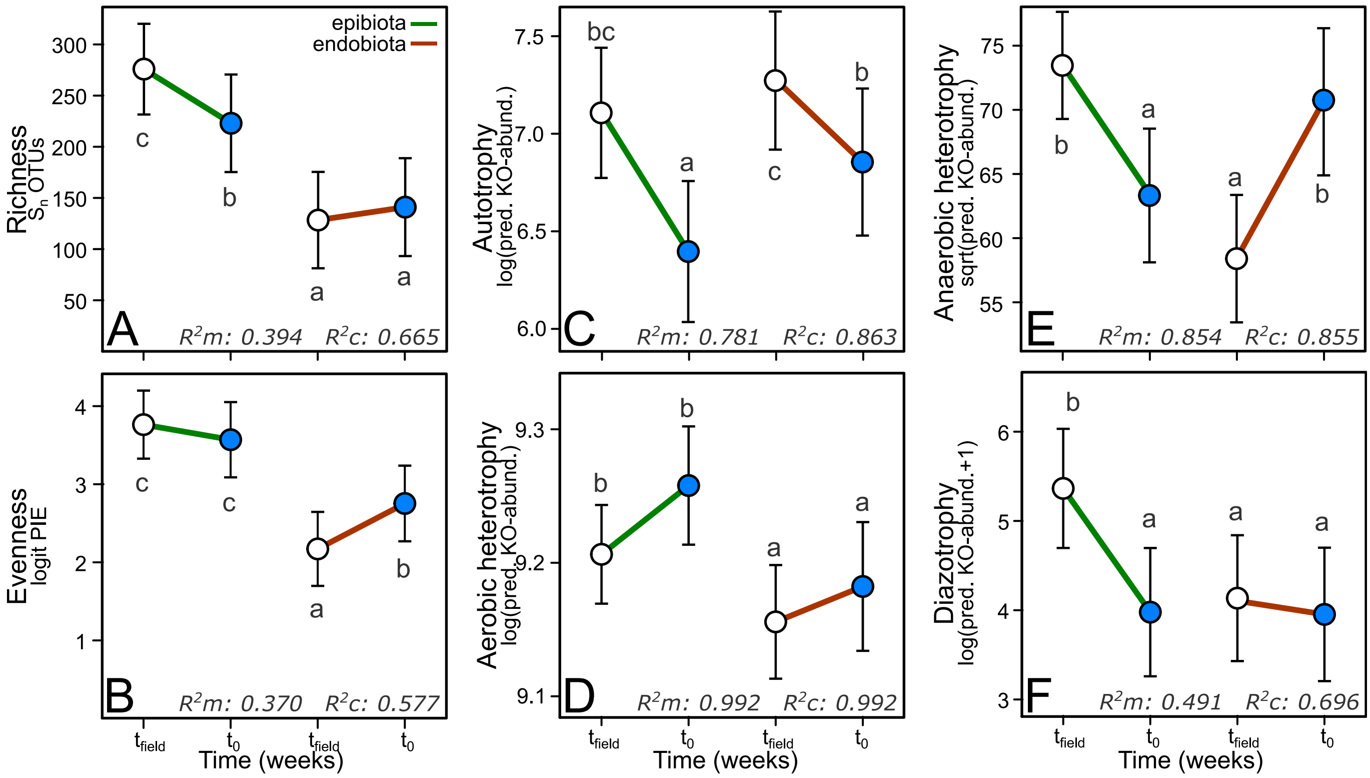

Figure S2. Changes in community properties between the field and start of the experiment. Panels display diversity changes in (A: rarefied OTU richness; Sn) and (B: evenness). Predicted functional groups are displayed in C-F (autotrophy, aerobic heterotrophy, anaerobic heterotrophy and diazotrophy, respectively)

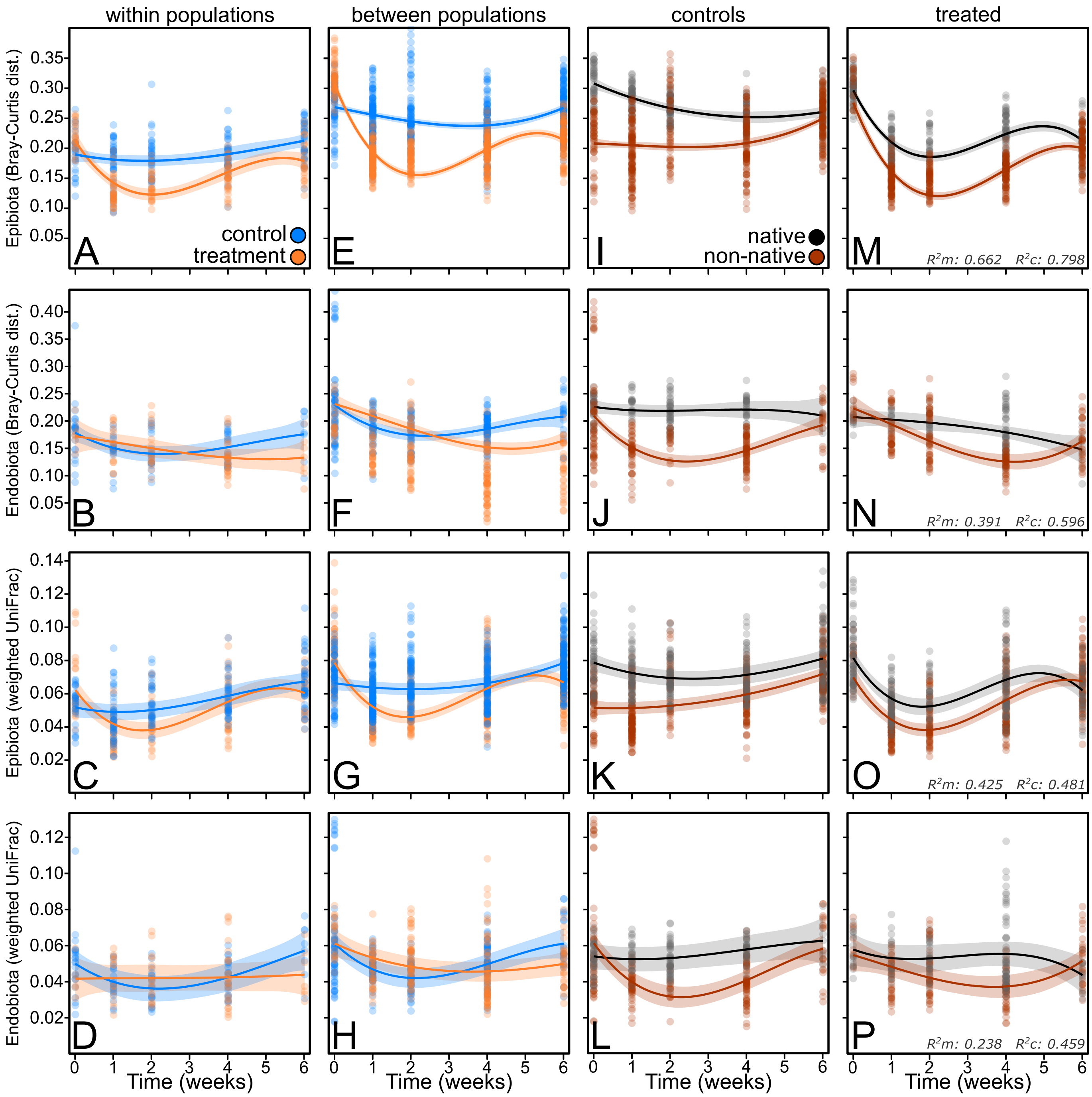

Figure S3. Regression curves of beta-diversity in terms of Bray-Curtis and weighted UniFrac distances. Diagrams display the mean distance within populations (controls in blue and treated algae in orange; A-D) and between populations (E-H) over time, between native (black) and non-native (red) controls over time (I-L) and between native and nonnative treated individuals over time (M-P). The 95% confidence regions are indicated in shades of the corresponding color. Marginal and conditional R^2 values of the models are displayed in the bottom right corner of the most right panel corresponding to the same model.

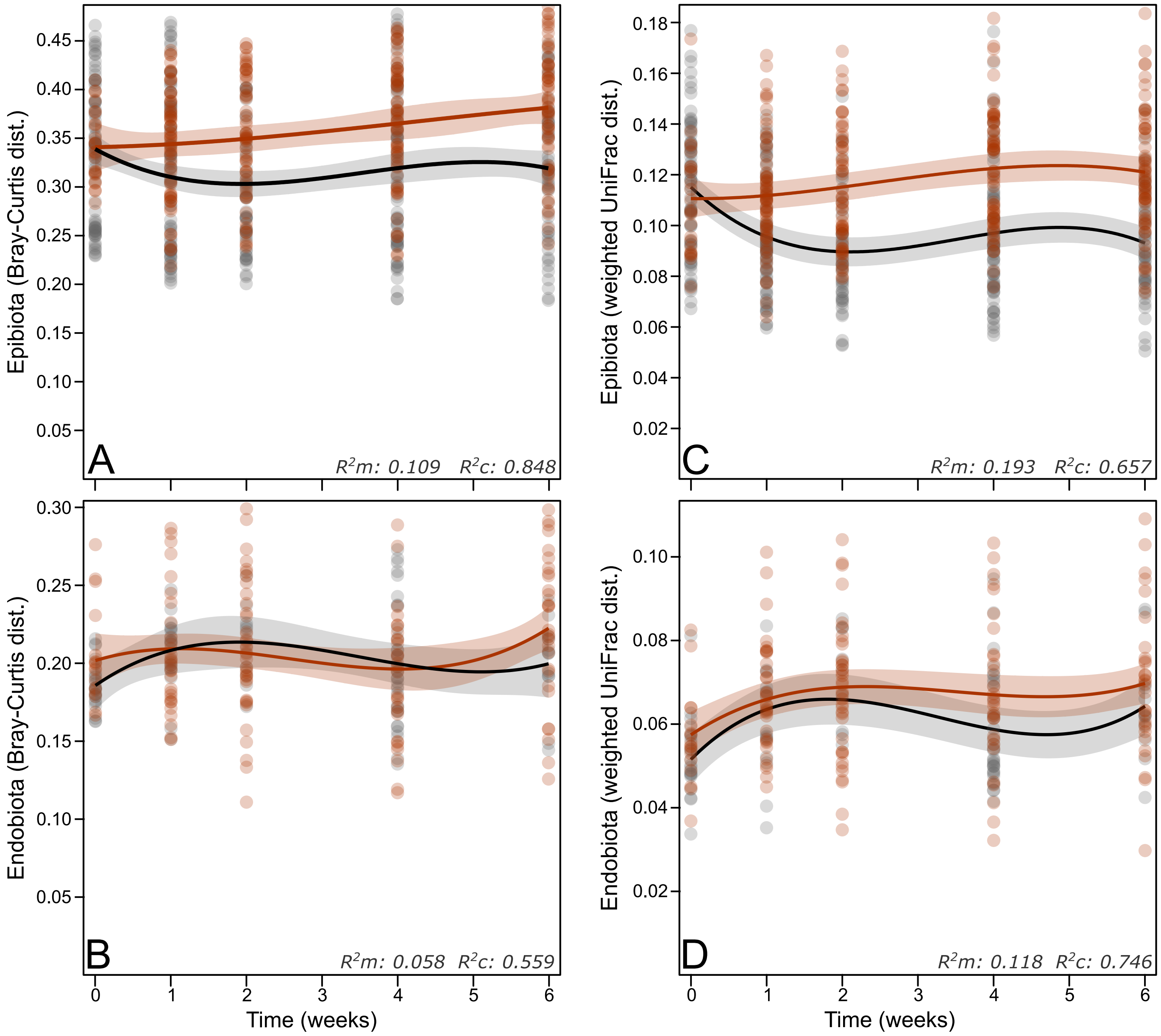

Figure S4. Regression curves of Bray-Curtis (A, B) and weighted UniFrac distances (C, D), within epibiota (A, C) and endobtioa (B, D) with respect to the field. Diagrams display the mean distance with respect to microbiota in the field for treated native (black) and treated non-native (red) holobionts over time. The 95% confidence regions are indicated in shades of the corresponding color. Marginal and conditional R^2 values of the models are displayed in the bottom right corner.

Table S1. Summary of samples remaining after data quality treatment and included in analyses.

										samples included in analysis[5]											
										endo						*epi*					
code	range	coast[1]	eco-region[2]	country	population[3]	longitude	latitude	collection date	collectors[4]	t_f	t_0	t_1	t_2	t_4	t_6	t_f	t_0	t_1	t_2	t_4	t_6
sou1	native	WP	NH	Japan	sou	141°3'34.90"E	38°21'10.20"N	27-8-17	MN,FW,TB	–	–/–	C/–	C/T	–/T	–/T	x	–/T	C/T	C/T	C/T	–/T
sou2	native	WP	NH	Japan	sou	141°3'34.90"E	38°21'10.20"N	27-8-17	MN,FW,TB	x	–/–	–/–	–/T	–/T	–/–	x	C/T	C/T	C/T	C/T	C/–
sou5	native	WP	NH	Japan	sou	141°3'34.90"E	38°21'10.20"N	27-8-17	MN,FW,TB	–	C/T	C/T	–/–	C/T	C/T	x	C/T	C/T	C/T	C/T	C/T
sou6	native	WP	NH	Japan	sou	141°3'34.90"E	38°21'10.20"N	27-8-17	MN,FW,TB	x	–/–	–/–	C/T	–/–	–/–	x	–/T	–/T	C/T	C/T	C/–
sou4	native	WP	NH	Japan	sou	141°3'34.90"E	38°21'10.20"N	27-8-17	MN,FW,TB	–						x					
sou8	native	WP	NH	Japan	sou	141°3'34.90"E	38°21'10.20"N	27-8-17	MN,FW,TB	x						x					
akk4	native	WP	OC	Japan	akk	144°56'59.30"E	43°2'51.90"N	31-8-17	MN,TB	–	C/T	C/T	C/T	–/–	–/–	x	C/T	C/T	C/–	C/T	C/T
akk7	native	WP	OC	Japan	akk	144°56'59.30"E	43°2'51.90"N	31-8-17	MN,TB	–	C/–	–/T	C/T	C/T	–/T	x	C/T	C/T	C/–	C/T	C/T
akk8	native	WP	OC	Japan	akk	144°56'59.30"E	43°2'51.90"N	31-8-17	MN,TB	–	C/T	C/T	C/T	C/T	–/T	x	C/T	C/T	C/–	C/T	C/T
akk9	native	WP	OC	Japan	akk	144°56'59.30"E	43°2'51.90"N	31-8-17	MN,TB	–	C/–	–/T	C/T	C/T	–/–	x	C/T	C/T	C/–	C/T	C/T
akk1	native	WP	OC	Japan	akk	144°56'59.30"E	43°2'51.90"N	31-8-17	MN,TB	–						x					
akk10	native	WP	OC	Japan	akk	144°56'59.30"E	43°2'51.90"N	31-8-17	MN,TB	–						x					
ron3	native	WP	YS	China	ron	122°20'44.60"E	37°6'45.70"N	1-9-17	GW,FW	x	C/T	C/–	C/–	–/T	–/–	x	C/T	C/T	C/T	C/T	C/T
ron4	native	WP	YS	China	ron	122°20'44.60"E	37°6'45.70"N	1-9-17	GW,FW	x	C/T	C/–	C/–	C/T	C/–	x	C/T	C/T	C/T	C/T	C/T
ron5	native	WP	YS	China	ron	122°20'44.60"E	37°6'45.70"N	1-9-17	GW,FW	x	C/T	C/T	C/–	C/T	C/–	x	C/T	C/T	C/T	C/T	C/T
ron8	native	WP	YS	China	ron	122°20'44.60"E	37°6'45.70"N	1-9-17	GW,FW		C/–	C/T	C/–	C/T	C/T		–/T	C/T	C/T	C/T	C/–
ron6	native	WP	YS	China	ron	122°20'44.60"E	37°6'45.70"N	1-9-17	GW,FW	x						x					
ron9	native	WP	YS	China	ron	122°20'44.60"E	37°6'45.70"N	1-9-17	GW,FW	x						x					
fdm4	nonnative	EA	CS	France	fdm	1°58'11.30"W	48°30'52.60"N	21-9-17	MV,FW	–	C/T	C/T	–/T	C/T	C/T	x	–/T	C/T	–/T	C/T	C/T
fdm5	nonnative	EA	CS	France	fdm	1°58'11.30"W	48°30'52.60"N	21-9-17	MV,FW		C/T	C/T	C/T	C/–	–/–		–/–	C/T	–/–	C/T	C/T
fdm6	nonnative	EA	CS	France	fdm	1°58'11.30"W	48°30'52.60"N	21-9-17	MV,FW	x	–/–	C/T	–/–	C/T	C/T	x	–/–	C/T	–/–	C/–	C/T
fdm9	nonnative	EA	CS	France	fdm	1°58'11.30"W	48°30'52.60"N	21-9-17	MV,FW	–	–/–	–/–	–/–	–/–	–/–	x	–/–	C/T	–/–	–/T	C/–
fdm1	nonnative	EA	CS	France	fdm	1°58'11.30"W	48°30'52.60"N	21-9-17	MV,FW	–						x					
fdm10	nonnative	EA	CS	France	fdm	1°58'11.30"W	48°30'52.60"N	21-9-17	MV,FW	x						–					
nor2	nonnative	EA	NS	Germany	nor	8°48'44.65"E	54°29'9.34"N	11-9-17	FW,NS	–	C/–	–/T	–/T	C/T	–/–	x	C/–	C/–	C/T	C/T	C/T
nor4	nonnative	EA	NS	Germany	nor	8°48'44.65"E	54°29'9.34"N	11-9-17	FW,NS	–	C/T	–/T	–/T	–/T	–/–	x	C/–	C/T	C/T	C/T	C/T
nor5	nonnative	EA	NS	Germany	nor	8°48'44.65"E	54°29'9.34"N	11-9-17	FW,NS	x	–/–	–/–	–/–	C/T	C/T	x	–/–	C/T	C/T	C/–	C/–
nor6	nonnative	EA	NS	Germany	nor	8°48'44.65"E	54°29'9.34"N	11-9-17	FW,NS	x	C/–	–/–	–/–	C/–	C/–	x	C/–	C/T	C/T	–/T	C/T
nor1	nonnative	EA	NS	Germany	nor	8°48'44.65"E	54°29'9.34"N	11-9-17	FW,NS	x						x					
nor8	nonnative	EA	NS	Germany	nor	8°48'44.65"E	54°29'9.34"N	11-9-17	FW,NS	–						x					
tmb84	nonnative	EP	NC	California	tmb	121°45'14.098"W	36°50'44.828"N	21-9-17	SAKH,GB	x						x					
tmb72	nonnative	EP	NC	California	tmb	121°45'14.098"W	36°50'44.828"N	21-9-17	SAKH,GB		C/–	C/T	C/T	C/–	C/T		C/T	C/T	C/T	C/T	C/T
tmb73	nonnative	EP	NC	California	tmb	121°45'14.098"W	36°50'44.828"N	21-9-17	SAKH,GB		C/T	C/T	C/–	C/–	–/–		C/T	C/T	–/T	C/T	C/T
tmb85	nonnative	EP	NC	California	tmb	121°45'14.098"W	36°50'44.828"N	21-9-17	SAKH,GB		C/–	C/T	C/T	–/–	C/–		C/–	C/T	–/T	C/T	C/T
tmb75	nonnative	EP	NC	California	tmb	121°45'14.098"W	36°50'44.828"N	21-9-17	SAKH,GB	x	C/T	C/T	C/T	C/–	C/T	x	C/T	C/T	C/T	C/T	C/T
tmb17	nonnative	EP	NC	California	tmb	121°45'14.098"W	36°50'44.828"N	21-9-17	SAKH,GB	x						x					

Table S1. Continued

										samples included in analysis[5]											
										endo						*epi*					
code	range	coast[1]	eco-region[2]	country	popu-lation[3]	longitude	latitude	collec-tion date	collec-tors[4]	t_f	t_0	t_1	t_2	t_4	t_6	t_f	t_0	t_1	t_2	t_4	t_6
tmb76	nonnative	EP	NC	California	tmb	121°45'14.098"W	36°50'44.828"N	21-9-17	SAKH,GB	x						x					
tmb74	nonnative	EP	NC	California	tmb	121°45'14.098"W	36°50'44.828"N	21-9-17	SAKH,GB	x						x					
ccb30	nonnative	WA	V	Virginia	ccb	76°1'21.875"W	37°16'19.114"N	9-9-17	SAKH,GB	x	-/-	C/-	-/T	-/T	-/T	-	C/-	C/-	C/T	C/T	C/T
ccb32	nonnative	WA	V	Virginia	ccb	76°1'21.875"W	37°16'19.114"N	9-9-17	SAKH,GB		C/-	C/-	-/T	C/T	-/T		C/T	C/T	C/-	C/T	C/T
ccb46	nonnative	WA	V	Virginia	ccb	76°1'21.875"W	37°16'19.114"N	9-9-17	SAKH,GB		-/-	-/-	-/T	C/T	-/-		C/T	C/T	C/T	C/T	-/-
ccb50	nonnative	WA	V	Virginia	ccb	76°1'21.875"W	37°16'19.114"N	9-9-17	SAKH,GB	x	C/-	C/-	C/-	C/T	-/T	x	C/-	C/T	C/T	C/T	C/T
ccb33	nonnative	WA	V	Virginia	ccb	76°1'21.875"W	37°16'19.114"N	9-9-17	SAKH,GB	x						-					
ccb7a	nonnative	WA	V	Virginia	ccb	76°1'21.875"W	37°16'19.114"N	9-9-17	SAKH,GB	x						x					
ccb3	nonnative	WA	V	Virginia	ccb	76°1'21.875"W	37°16'19.114"N	9-9-17	SAKH,GB	-						x					

[1] *Abbreviations for continental coasts: Western Pacific (WP), Eastern Pacific (EP), Western Atlantic (WA) and Eastern Atlantic (EA)*

[2] *Abbreviations for ecoregions: Northeastern Honshu (NH), Oyashio Current (OC), Yeallow Sea (YS), Celtic Seas (CS), North Sea (NS), Northern California (NC) and Virginian (V)*

[3] *Abbreviations for populations: Soukanzan (sou), Akkeshi (akk), Rongcheng (ron), Pleudihen-sur-Rance (fdm), Nordstrand (nor), Tomales Bay (tmb), Cape Charles Beach (ccb)*

[4] *Colllectors are abbreviated by author initials.*

[5] *Crosses indicate field samples with more than 1000 after quality filtering included in the analyses. Samples taken during the experiment and passing the quality filtration steps are displayed as control/treatment. Dashed indicate samples not passing quality criteria whereas samples not included in the field study or experiment are represented by empty cells*

Table S2. Schematic overview of the experiment.

**day**	**group 1:** sou	**group 2:** akk & ron	**group 3:** ccb	**group: 4** nor	**group: 5** fdm & tmb	**date**
1	field collection sou (**t_f**)					27-08-2017
2						28-08-2017
3						29-08-2017
4						30-08-2017
5	start acclimation	field collection akk (**t_f**)				31-08-2017
6		field collection ron (**t_f**)				01-09-2017
7						02-09-2017
8						03-09-2017
9						04-09-2017
10						05-09-2017
11		start acclimation				06-09-2017
12	water exchange					07-09-2017
13						08-09-2017
14			field collection ccb (**t_f**)			09-09-2017
15						10-09-2017
16				field collection nor (**t_f**)		11-09-2017
17						12-09-2017
18		water exchange	start acclimation			13-09-2017
19	water exchange					14-09-2017
20				start acclimation		15-09-2017
21						16-09-2017
22						17-09-2017
23						18-09-2017
24						19-09-2017
25		water exchange	water exchange			20-09-2017
26	water exchange				field collection fdm & tmb (**t_f**)	21-09-2017
27				water exchange		22-09-2017
28						23-09-2017
29						24-09-2017
30						25-09-2017
31					start acclimation	26-09-2017
32		water exchange	water exchange			27-09-2017
33	water exchange					28-09-2017
34						29-09-2017
35				water exchange		30-09-2017
36						01-10-2017
37	**start treatment**					02-10-2017
38					water exchange	03-10-2017
39		water exchange	water exchange			04-10-2017
40	end treatment (**t_0**)					05-10-2017
41						06-10-2017
42				water exchange		07-10-2017

Table S2. Schematic overview of the experiment. (continued)

day	group 1: sou	group 2: akk & ron	group 3: ccb	group: 4 nor	group: 5 fdm & tmb	date
43		**start treatment**				08-10-2017
44						09-10-2017
45			water exchange		water exchange	10-10-2017
46	water exchange	end treatment (t_0)				11-10-2017
47	sampling (t_1)					12-10-2017
48						13-10-2017
49			**start treatment**			14-10-2017
50						15-10-2017
51						16-10-2017
52		water exchange	end treatment (t_0)	**start treatment**	water exchange	17-10-2017
53	water exchange	sampling (t_1)				18-10-2017
54	sampling (t_2)					19-10-2017
55				end treatment (t_0)		20-10-2017
56						21-10-2017
57						22-10-2017
58			water exchange		water exchange	23-10-2017
59		water exchange	sampling (t_1)			24-10-2017
60	water exchange	sampling (t_2)				25-10-2017
61				water exchange		26-10-2017
62				sampling (t_1)	**start treatment**	27-10-2017
63						28-10-2017
64						29-10-2017
65			water exchange		end treatment (t_0)	30-10-2017
66		water exchange	sampling (t_2)			31-10-2017
67	water exchange					01-11-2017
68	sampling (t_4)			water exchange		02-11-2017
69				sampling (t_2)		03-11-2017
70						04-11-2017
71					water exchange	05-11-2017
72			water exchange		sampling (t_1)	06-11-2017
73		water exchange				07-11-2017
74	water exchange	sampling (t_4)				08-11-2017
75				water exchange		09-11-2017
76						10-11-2017
77						11-11-2017
78					water exchange	12-11-2017
79			water exchange		sampling (t_2)	13-11-2017
80		water exchange	sampling (t_4)			14-11-2017
81	sampling (t_6)					15-11-2017
82				water exchange		16-11-2017
83				sampling (t_4)		17-11-2017
84						18-11-2017

Table S2. Schematic overview of the experiment. (continued)

day	group 1: sou	group 2: akk & ron	group 3: ccb	group: 4 nor	group: 5 fdm & tmb	date
85					water exchange	19-11-2017
86			water exchange			20-11-2017
87		sampling ($\mathbf{t_6}$)				21-11-2017
88						22-11-2017
89				water exchange		23-11-2017
90						24-11-2017
91						25-11-2017
92					water exchange	26-11-2017
93			sampling ($\mathbf{t_6}$)		sampling ($\mathbf{t_4}$)	27-11-2017
94						28-11-2017
95						29-11-2017
96						30-11-2017
97				sampling ($\mathbf{t_6}$)		01-12-2017
98						02-12-2017
99					water exchange	03-12-2017
100						04-12-2017
101						05-12-2017
102						06-12-2017
103						07-12-2017
104						08-12-2017
105						09-12-2017
106						10-12-2017
107					sampling ($\mathbf{t_6}$)	11-12-2017

Group 1 (in red; Soukanzan: sou), group 2 (in yellow; Akkeshi & Rongcheng: akk & ron), group 3 (in blue; Cape Charles Beach: ccb), group 4 (in pink: Nordstrand: nor) and group 5 (Pleudihen-sur-Rance & Tomales Bay: fdm & tmb). The days during which the treated algae were exposed to the antibiotic mix are marked in black. Sampling moments are labelled with t_f (in the field) t_0, t_1, t_2, t_4 and t_6. Right before the application of the treatment and during the rest of the experiment, wet weight was recorded with every water exchange.

Table S4. Core OTUs identified in this study by cross comparison to Bonthond et al. (2020).

core	core OTU in Bonthond et al. (2020)	OTU in this study	Δ bp
endo	core_Otu00002	Otu00002	0
epi	core_Otu00202	Otu00003	0
alga	core_Otu00003	Otu00007	0
alga	core_Otu00006	Otu00008	0
alga	core_Otu00017	Otu00009	0
endo	core_Otu00005	Otu00010	0
alga	core_Otu00004	Otu00014	0
epi	core_Otu00037	Otu00015	0
alga	core_Otu00040	Otu00019	0
epi	core_Otu00146	Otu00021	0
alga	core_Otu00001	Otu00022	0
endo	core_Otu00012	Otu00024	0
alga	core_Otu00013	Otu00026	0
endo	core_Otu00007	Otu00031	0
epi	core_Otu00061	Otu00035	0
epi	core_Otu00025	Otu00037	0
alga	core_Otu00070	Otu00038	0
alga	core_Otu00477	Otu00040	0
alga	core_Otu00014	Otu00041	0
alga	core_Otu00018	Otu00043	0
alga	core_Otu00015	Otu00049	0
epi	core_Otu00019	Otu00054	0
alga	core_Otu00010	Otu00055	0
alga	core_Otu00069	Otu00057	0
epi	core_Otu00027	Otu00064	0
alga	core_Otu00024	Otu00069	0
alga	core_Otu00028	Otu00073	0
epi	core_Otu00030	Otu00074	0
epi	core_Otu00044	Otu00077	0
alga	core_Otu00078	Otu00079	0
epi	core_Otu00076	Otu00081	0
endo	core_Otu00082	Otu00084	0
epi	core_Otu01112	Otu00086	0
alga	core_Otu00016	Otu00088	0
epi	core_Otu00059	Otu00090	0
epi	core_Otu00166	Otu00093	0
epi	core_Otu00592	Otu00094	0
alga	core_Otu00057	Otu00095	0
endo	core_Otu00067	Otu00097	0
alga	core_Otu00390	Otu00098	0
epi	core_Otu00098	Otu00105	0
endo	core_Otu00147	Otu00106	0
alga	core_Otu00112	Otu00107	0
epi	core_Otu00021	Otu00109	0
epi	core_Otu00039	Otu00111	0
epi	core_Otu00064	Otu00113	0
endo	core_Otu00141	Otu00118	0
alga	core_Otu00008	Otu00119	0
alga	core_Otu00110	Otu00132	0
alga	core_Otu00063	Otu00138	0
endo	core_Otu00048	Otu00142	0
alga	core_Otu00303	Otu00146	0
epi	core_Otu00077	Otu00147	0
alga	core_Otu00091	Otu00150	0
alga	core_Otu00111	Otu00151	0
epi	core_Otu00068	Otu00160	0
epi	core_Otu00080	Otu00162	0
alga	core_Otu00104	Otu00163	0
alga	core_Otu00148	Otu00164	0
alga	core_Otu00055	Otu00166	0
endo	core_Otu00041	Otu00174	0
epi	core_Otu00058	Otu00177	0

Table S4. Continued

epi	core_Otu00065	Otu00178	0
alga	core_Otu00286	Otu00180	0
endo	core_Otu00086	Otu00184	0
epi	core_Otu00949	Otu00187	0
alga	core_Otu00109	Otu00195	0
endo	core_Otu00236	Otu00199	0
endo	core_Otu00193	Otu00203	0
alga	core_Otu00150	Otu00204	0
alga	core_Otu00383	Otu00206	0
alga	core_Otu00066	Otu00212	0
alga	core_Otu00174	Otu00219	0
alga	core_Otu00574	Otu00224	0
epi	core_Otu00103	Otu00241	0
epi	core_Otu00960	Otu00246	0
alga	core_Otu00169	Otu00247	0
alga	core_Otu00128	Otu00248	0
alga	core_Otu00134	Otu00256	0
alga	core_Otu00154	Otu00257	0
epi	core_Otu00087	Otu00258	0
epi	core_Otu00094	Otu00262	0
alga	core_Otu00182	Otu00272	0
epi	core_Otu00546	Otu00281	0
epi	core_Otu00270	Otu00286	0
alga	core_Otu00323	Otu00290	0
epi	core_Otu00649	Otu00293	0
epi	core_Otu00235	Otu00295	0
alga	core_Otu00413	Otu00301	0
endo	core_Otu00140	Otu00312	0
alga	core_Otu00149	Otu00316	0
alga	core_Otu00516	Otu00321	0
alga	core_Otu00137	Otu00322	0
epi	core_Otu00356	Otu00325	0
epi	core_Otu00221	Otu00326	0
epi	core_Otu00168	Otu00328	0
alga	core_Otu00117	Otu00332	0
alga	core_Otu00219	Otu00333	0
alga	core_Otu00187	Otu00335	0
alga	core_Otu00675	Otu00343	0
alga	core_Otu00304	Otu00354	0
endo	core_Otu00339	Otu00359	0
epi	core_Otu00184	Otu00363	0
endo	core_Otu00319	Otu00377	0
alga	core_Otu00225	Otu00381	0
alga	core_Otu00344	Otu00388	0
alga	core_Otu00172	Otu00389	0
alga	core_Otu00549	Otu00408	0
alga	core_Otu00294	Otu00419	0
alga	core_Otu00460	Otu00421	0
alga	core_Otu00326	Otu00433	0
alga	core_Otu01267	Otu00435	0
endo	core_Otu00347	Otu00437	0
epi	core_Otu00259	Otu00439	0
epi	core_Otu00643	Otu00452	0
alga	core_Otu00283	Otu00460	0
alga	core_Otu00454	Otu00477	0
alga	core_Otu00277	Otu00495	0
alga	core_Otu00288	Otu00496	0
epi	core_Otu00586	Otu00504	0
epi	core_Otu00375	Otu00527	0
epi	core_Otu00512	Otu00539	0
alga	core_Otu00204	Otu00561	0
alga	core_Otu00622	Otu00578	0
epi	core_Otu00686	Otu00580	0

Table S4. Continued

alga	core_Otu00991	Otu00586	0
alga	core_Otu00291	Otu00598	0
epi	core_Otu00228	Otu00606	0
epi	core_Otu00439	Otu00625	0
alga	core_Otu00485	Otu00634	0
alga	core_Otu00285	Otu00635	0
alga	core_Otu00536	Otu00655	0
alga	core_Otu01057	Otu00658	0
epi	core_Otu00382	Otu00659	0
epi	core_Otu03335	Otu00674	0
alga	core_Otu00994	Otu00697	0
epi	core_Otu00295	Otu00709	0
epi	core_Otu00320	Otu00730	0
endo	core_Otu00783	Otu00733	0
alga	core_Otu00504	Otu00740	0
alga	core_Otu00669	Otu00776	0
epi	core_Otu00412	Otu00799	0
epi	core_Otu00522	Otu00801	0
epi	core_Otu03089	Otu00807	0
alga	core_Otu00814	Otu00811	0
epi	core_Otu00640	Otu00814	0
epi	core_Otu00275	Otu00825	0
alga	core_Otu00691	Otu00833	0
alga	core_Otu00445	Otu00838	0
alga	core_Otu00613	Otu00875	0
alga	core_Otu00380	Otu00920	0
epi	core_Otu00679	Otu00923	0
epi	core_Otu00537	Otu00941	0
epi	core_Otu00583	Otu00979	0
epi	core_Otu00612	Otu01000	0
alga	core_Otu00937	Otu01010	0
epi	core_Otu00774	Otu01019	0
epi	core_Otu00690	Otu01038	0
epi	core_Otu01126	Otu01047	0
epi	core_Otu00557	Otu01094	0
epi	core_Otu00972	Otu01141	0
endo	core_Otu00970	Otu01151	0
epi	core_Otu00695	Otu01158	0
epi	core_Otu01312	Otu01166	0
alga	core_Otu00563	Otu01173	0
epi	core_Otu00391	Otu01174	0
epi	core_Otu00654	Otu01178	0
epi	core_Otu00476	Otu01217	0
alga	core_Otu00573	Otu01250	0
epi	core_Otu01553	Otu01254	0
epi	core_Otu00876	Otu01255	0
alga	core_Otu01398	Otu01266	0
epi	core_Otu00466	Otu01269	0
epi	core_Otu01006	Otu01328	0
epi	core_Otu00934	Otu01355	0
alga	core_Otu00580	Otu01362	0
epi	core_Otu00651	Otu01369	0
endo	core_Otu01181	Otu01387	0
alga	core_Otu01208	Otu01490	0
endo	core_Otu01365	Otu01527	0
epi	core_Otu01292	Otu01606	0
alga	core_Otu01128	Otu01607	0
epi	core_Otu00595	Otu01653	0
epi	core_Otu01150	Otu01738	0
alga	core_Otu01331	Otu01839	0
epi	core_Otu01119	Otu01878	0
epi	core_Otu00384	Otu01884	0
epi	core_Otu01249	Otu01987	0

Table S4. Continued

alga	core_Otu00195	Otu02072	0
alga	core_Otu01634	Otu02096	0
epi	core_Otu01076	Otu02127	0
epi	core_Otu00562	Otu02147	0
endo	core_Otu01796	Otu02165	0
alga	core_Otu02433	Otu02311	0
alga	core_Otu02051	Otu02314	0
epi	core_Otu01325	Otu02323	0
epi	core_Otu01728	Otu02400	0
alga	core_Otu02469	Otu02408	0
epi	core_Otu00936	Otu02436	0
epi	core_Otu01474	Otu02450	0
alga	core_Otu01261	Otu02473	0
epi	core_Otu01529	Otu02571	0
epi	core_Otu01772	Otu02613	0
alga	core_Otu01218	Otu02882	0
epi	core_Otu02486	Otu02895	0
epi	core_Otu02197	Otu03205	0
epi	core_Otu00740	Otu03274	0
epi	core_Otu02052	Otu03354	0
alga	core_Otu03263	Otu03618	0
epi	core_Otu02087	Otu03646	0
epi	core_Otu02863	Otu03828	0
epi	core_Otu03011	Otu03893	0
epi	core_Otu02067	Otu03916	0
epi	core_Otu03326	Otu03920	0
epi	core_Otu02759	Otu04080	0
alga	core_Otu02610	Otu04452	0
epi	core_Otu03076	Otu05089	0
endo	core_Otu02787	Otu08149	0
alga	core_Otu00020	Otu00016	3
alga	core_Otu00023	Otu00029	2
alga	core_Otu00034	Otu06204	3
alga	core_Otu00035	Otu06508	10
alga	core_Otu00052	Otu00123	2
alga	core_Otu00083	Otu00709	6
alga	core_Otu00089	Otu00251	1
alga	core_Otu00130	Otu00044	4
alga	core_Otu00133	Otu00126	2
alga	core_Otu00153	Otu01039	5
epi	core_Otu00155	Otu00709	10
epi	core_Otu00179	Otu01189	3
epi	core_Otu00197	Otu00059	1
epi	core_Otu00199	Otu00727	6
epi	core_Otu00200	Otu00340	1
epi	core_Otu00216	Otu00912	6
epi	core_Otu00218	Otu00337	3
epi	core_Otu00230	Otu00273	1
alga	core_Otu00242	Otu04147	2
epi	core_Otu00245	Otu00413	6
epi	core_Otu00262	Otu06481	14
alga	core_Otu00269	Otu02079	1
epi	core_Otu00280	Otu00760	7
epi	core_Otu00290	Otu01757	3
epi	core_Otu00298	Otu00624	1
alga	core_Otu00300	Otu00577	2
alga	core_Otu00301	Otu00534	1
epi	core_Otu00322	Otu00837	2
epi	core_Otu00332	Otu00900	1
epi	core_Otu00343	Otu07111	6
epi	core_Otu00370	Otu00405	4
alga	core_Otu00387	Otu01104	6
epi	core_Otu00406	Otu00670	4

Table S4. Continued

epi	core_Otu00416	Otu00125	3
epi	core_Otu00421	Otu00230	1
epi	core_Otu00422	Otu01193	1
epi	core_Otu00441	Otu03376	5
alga	core_Otu00446	Otu00259	5
alga	core_Otu00461	Otu00369	3
endo	core_Otu00468	Otu00445	4
epi	core_Otu00470	Otu07593	3
epi	core_Otu00568	Otu00271	10
alga	core_Otu00633	Otu03661	6
epi	core_Otu00652	Otu00509	1
alga	core_Otu00658	Otu00884	3
epi	core_Otu00684	Otu00693	1
epi	core_Otu00731	Otu00633	22
alga	core_Otu00749	Otu00278	4
alga	core_Otu00755	Otu03833	3
epi	core_Otu00766	Otu01660	9
epi	core_Otu00784	Otu00246	2
epi	core_Otu00791	Otu00831	6
epi	core_Otu00902	Otu04165	4
epi	core_Otu01105	Otu01592	1
epi	core_Otu01289	Otu00909	1
epi	core_Otu01299	Otu03245	2
epi	core_Otu01412	Otu01200	4
epi	core_Otu01426	Otu01929	3
alga	core_Otu01454	Otu01702	2
epi	core_Otu01514	Otu02302	3
epi	core_Otu01539	Otu00091	2
alga	core_Otu01544	Otu03231	2
endo	core_Otu01585	Otu02358	2
epi	core_Otu01594	Otu02246	2
epi	core_Otu02159	Otu02808	7
alga	core_Otu02183	Otu00580	5
alga	core_Otu02188	Otu06067	4
epi	core_Otu02221	Otu01310	3
epi	core_Otu02388	Otu04680	1
endo	core_Otu02399	Otu01298	6
alga	core_Otu02654	Otu06117	1

Core types include epi-, endophytic and alga (both epi- and endophytic). OTU numbers and corresponding nucleotide sequences from both studies are shown with the number of nucleotide differences.

Table S3. Statistical output of models fitted in this study. (A) Core abundance

model structure	(core/size) ~ substrate * treatment * range + (1|population) + (1|individual)							
		Sum Sq	Mean Sq	NumDF	DenDF	F value	Pr(>F)	
ANOVA table	substrate	0.762	0.762	1	115.20	59.763	**4.39E-12**	***
	treatment	3.215	1.608	2	114.78	126.018	**1.10E-29**	***
	range	0.003	0.003	1	5.12	0.258	0.633	
	substrate:treatment	0.068	0.034	2	114.51	2.653	0.075	.
	substrate:range	0.036	0.036	1	115.20	2.848	0.094	.
	treatment:range	0.036	0.018	2	114.78	1.412	0.248	
	substrate:treatment:range	0.016	0.008	2	114.51	0.629	0.535	
			Estimate	Std. Error	df	t value	Pr(<|t|)	
pairwise comparisons	field – treatmentC		0.364	0.026	114.2	13.965	**< 2.2E-16**	***
	field – treatmentT		0.308	0.026	115.1	11.904	**< 2.2E-16**	***
	treatmentC – treatmentT		-0.056	0.030	115.1	-1.895	0.06	.

*Model structures are written in the syntax of the R package lme4 (Bates et al. 2015). The natural logarithm of the sequencing depth is abbreviated with LSD. P-values < 0.05 are displayed in bold and with stars (. < 0.1, * < 0.05, ** < 0.01 and *** < 0.001).*

Table S3. Statistical output of models fitted in this study. **(B)** Host relative growth rate

model structure	~ range * treatment * poly(time,3) + (1|population) + (1|individual)							
		Sum Sq	Mean Sq	NumDF	DenDF	F value	Pr(>F)	
ANOVA table	range	1665.0	1665.0	1	4.88	7.437	**0.043**	*
	poly(time,3)	9609.3	3203.1	3	364.02	14.307	**7.84E-09**	***
	treatment	439.8	439.8	1	362.86	1.964	0.162	
	range:poly(time,3)	3312.0	1104.0	3	364.02	4.931	**2.28E-03**	**
	range:treatment	0.0	0.0	1	362.86	0.000	0.998	
	poly(time,3):treatment	462.7	154.2	3	362.86	0.689	0.559	
	range:poly(time,3):treatment	147.8	49.3	3	362.86	0.220	0.883	

*Model structures are written in the syntax of the R package lme4 (Bates et al. 2015). The natural logarithm of the sequencing depth is abbreviated with LSD. P-values < 0.05 are displayed in bold and with stars (. < 0.1, * < 0.05, ** < 0.01 and *** < 0.001).*

Table S3. Statistical output of models fitted in this study. **(C)** α-diversity

model structure: ~ timepoint + substrate + timepoint:substrate + (1|population) + (1|individual)

Sn — ANOVA table

	Sum Sq	Mean Sq	NumDF	DenDF	F value	Pr(>F)	
timepoint	9300	9300	1	90.687	2.670	0.106	
substrate	302685	302685	3	90.586	86.902	**6.95E-15**	***
timepoint:substrate	24499	24499	1	90.835	7.034	**9.44E-03**	**

Sn — Pairwise comparisons

	Estimate	Std. Error	df	t value	Pr(>t)	
tf:endo – t0:endo	-12.664	18.416	90.7	-0.688	0.493	
tf:endo – tf:epi	-147.589	16.145	90.8	-9.142	**1.62E-14**	***
tf:endo – t0:epi	-94.618	18.215	90.9	-5.195	**1.25E-06**	***
t0:endo – tf:epi	-134.925	16.602	90.4	-8.127	**2.18E-12**	***
t0:endo – t0:epi	-81.954	18.675	90.6	-4.388	**3.08E-05**	***
tf:epi – t0:epi	52.921	16.473	90.8	3.216	**1.80E-03**	**

logit PIE — ANOVA table

	Sum Sq	Mean Sq	NumDF	DenDF	F value	Pr(>F)	
timepoint	0.886	0.886	1	91.024	1.766	0.187	
substrate	33.357	33.357	1	90.878	68.066	**1.17E-12**	***
timepoint:substrate	3.424	3.424	1	91.239	6.988	**9.66E-03**	**

logit PIE — Pairwise comparisons

	Estimate	Std. Error	df	t value	Pr(>t)	
tf:endo – t0:endo	-0.582	0.218	91.1	-2.666	**9.08E-03**	**
tf:endo – tf:epi	-1.591	0.191	91.3	-8.320	**8.13E-13**	***
tf:endo – t0:epi	-1.398	0.216	91.3	-6.479	**4.60E-09**	***
t0:endo – tf:epi	-1.010	0.197	90.6	-5.129	**1.64E-06**	***
t0:endo – t0:epi	-0.816	0.221	90.9	-3.688	**3.84E-04**	***
tf:epi – t0:epi	0.193	0.195	91.1	0.990	0.325	

model structure: ~ poly(time,3) * substrate * treatment + (1|population) + (1|individual)

Sn — ANOVA table

	Sum Sq	Mean Sq	NumDF	DenDF	F value	Pr(>F)	
poly(time,3)	161833.771	53944.590	3	356.285	41.230	**6.80E-23**	***
substrate	92291.158	92291.158	1	357.220	70.539	**1.08E-15**	***
treatment	64671.504	64671.504	1	353.675	49.429	**1.07E-11**	***
poly(time,3):substrate	51131.954	17043.985	3	355.817	13.027	**4.32E-08**	***
poly(time,3):treatment	38312.435	12770.812	3	353.698	9.761	**3.34E-06**	***
substrate:treatment	14654.028	14654.028	1	354.586	11.200	**9.06E-04**	***
poly(time,3):substrate:treatment	17259.021	5753.007	3	353.881	4.397	**4.70E-03**	**

logit PIE — ANOVA table

	Sum Sq	Mean Sq	NumDF	DenDF	F value	Pr(>F)	
poly(time,3)	16.872	5.624	3	358.544	18.032	**6.40E-11**	***
substrate	23.498	23.498	1	359.049	75.339	**1.41E-16**	***
treatment	19.948	19.948	1	355.042	63.959	**1.81E-14**	***
poly(time,3):substrate	3.906	1.302	3	357.992	4.174	**6.35E-03**	**
poly(time,3):treatment	12.832	4.277	3	354.958	13.714	**1.75E-08**	***
substrate:treatment	1.644	1.644	1	356.207	5.271	**0.022**	*
poly(time,3):substrate:treatment	5.342	1.781	3	355.296	5.709	**7.95E-04**	***

*Model structures are written in the syntax of the R package lme4 (Bates et al. 2015). Abbreviations are used for natural logarithm of the sequencing depth (LSD), rarefied richness (Sn), probability of interspecific encounter (PIE). P-values < 0.05 are displayed in bold and with stars (. < 0.1, * < 0.05, ** < 0.01 and *** < 0.001).*

Table S3. Statistical output of models fitted in this study. **(D)** Functional groups

model structure: ~ timepoint + substrate + timepoint:substrate + (1|population) + (1|individual)

Response		Term	*Sum Sq*	*Mean Sq*	*NumDF*	*DenDF*	*F value*	*Pr(>F)*	
log(autotroph)	ANOVA table	LSD	59.053	59.053	1	94.419	246.121	**4.80E-28**	***
		timepoint	5.593	5.593	1	90.761	23.310	**5.56E-06**	***
		substrate	2.087	2.087	1	89.629	8.697	**4.07E-03**	**
		timepoint:substrate	0.488	0.488	1	89.576	2.032	0.157	
log(aerobic heterotroph)	ANOVA table	LSD	65.402	65.402	1	75.177	8180.492	**1.81E-78**	***
		timepoint	0.028	0.028	1	94.078	3.474	0.065	.
		substrate	0.087	0.087	1	92.059	10.896	**1.37E-03**	**
		timepoint:substrate	0.004	0.004	1	91.380	0.451	0.503	
			Sum Sq	Mean Sq	NumDF	DenDF	F value	Pr(>F)	
sqrt(anaerob ic heterotroph)	ANOVA table	LSD	85565.149	85565.149	1	40.512	396.813	**1.54E-22**	***
		timepoint	32.049	32.049	1	94.976	0.149	**0.701**	
		substrate	525.556	525.556	1	94.730	2.437	**0.122**	
		timepoint:substrate	4586.436	4586.436	1	93.556	21.270	**1.26E-05**	***
log(diazotrop h + 1)	ANOVA table	LSD	32.877	32.877	1	94.199	37.271	**2.28E-08**	***
		timepoint	10.794	10.794	1	90.937	12.236	**7.27E-04**	***
		substrate	8.430	8.430	1	89.967	9.556	**2.65E-03**	**
		timepoint:substrate	8.222	8.222	1	89.926	9.321	**2.98E-03**	**

model structure: ~ poly(time,3) * substrate * treatment + (1|population) + (1|individual)

Response		Term	Sum Sq	Mean Sq	NumDF	DenDF	F value	Pr(>F)	
log(autotroph)	ANOVA table	LSD	183.404	183.404	1	357.512	333.320	**4.40E-53**	***
		poly(time,3)	19.401	6.467	3	356.737	11.753	**2.33E-07**	***
		substrate	17.100	17.100	1	370.621	31.078	**4.78E-08**	***
		treatment	41.819	41.819	1	352.675	76.002	**1.13E-16**	***
		poly(time,3):substrate	1.373	0.458	3	355.323	0.832	0.477	
		poly(time,3):treatment	10.579	3.526	3	354.194	6.409	**3.08E-04**	***
		substrate:treatment	2.359	2.359	1	354.257	4.287	**0.039**	*
		poly(time,3):substrate:treatment	4.553	1.518	3	353.217	2.758	**0.042**	*
log(aerobic heterotroph)	ANOVA table	LSD	213.861	213.861	1	362.427	16913.021	**3.25E-306**	***
		poly(time,3)	0.044	0.015	3	357.883	1.147	0.330	
		substrate	0.005	0.005	1	373.691	0.389	0.533	
		treatment	0.015	0.015	1	354.038	1.218	0.270	
		poly(time,3):substrate	0.329	0.110	3	356.622	8.660	**1.46E-05**	***
		poly(time,3):treatment	0.110	0.037	3	355.913	2.892	**0.035**	*
		substrate:treatment	0.109	0.109	1	355.590	8.637	**0.004**	**
		poly(time,3):substrate:treatment	0.089	0.030	3	354.416	2.336	0.073	.

Table S3. Statistical output of models fitted in this study. **(D)** Functional groups (continued)

Model		Term	Sum Sq	Mean Sq	NumDF	DenDF	F value	Pr(>F)	
sqrt(anaerobic heterotroph)	ANOVA table	LSD	103564.419	103564.419	1	348.684	1055.300	**1.69E-107**	***
		poly(time,3)	2842.657	947.552	3	358.908	9.655	**3.82E-06**	***
		substrate	13.370	13.370	1	372.504	0.136	0.712	
		treatment	7300.525	7300.525	1	354.734	74.391	**2.17E-16**	***
		poly(time,3):substrate	717.194	239.065	3	357.524	2.436	0.065	.
		poly(time,3):treatment	2085.315	695.105	3	356.324	7.083	**1.23E-04**	***
		substrate:treatment	59.197	59.197	1	356.361	0.603	0.438	
		poly(time,3):substrate:treatment	608.010	202.670	3	355.281	2.065	0.105	
			Sum Sq	Mean Sq	NumDF	DenDF	F value	Pr(>F)	
log(diazotroph + 1)	ANOVA table	LSD	138.093	138.093	1	270.693	158.212	**7.01E-29**	***
		poly(time,3)	38.869	12.956	3	361.681	14.844	**3.91E-09**	***
		substrate	0.163	0.163	1	369.715	0.187	0.666	
		treatment	118.517	118.517	1	356.242	135.783	**8.36E-27**	***
		poly(time,3):substrate	6.674	2.225	3	360.147	2.549	0.056	.
		poly(time,3):treatment	35.251	11.750	3	357.767	13.462	**2.42E-08**	***
		substrate:treatment	3.465	3.465	1	358.171	3.969	**0.047**	*
		poly(time,3):substrate:treatment	1.149	0.383	3	357.309	0.439	0.725	

*Model structures are written in the syntax of the R packages lme4 (Bates et al. 2015). The natural logarithm of the sequencing depth is abbreviated with LSD. P-values < 0.05 are displayed in bold and with stars (. < 0.1, * < 0.05, ** < 0.01 and *** < 0.001).*

Table S3. Statistical output of models fitted in this study. **(E)** mGLMs

			Res.Df	Df.diff	Dev / val(F)	Pr(>dev) / Pr(>F)	
epibiota	mod I	community matrix ~ LSD + pop					
			Res.Df	Df.diff	Dev	Pr(>dev)	
	ANOVA table	(Intercept)	268	NA	NA	NA	
		LSD	267	1	25409.99	**0.002**	**
		population	261	6	77355.52	**0.002**	**
	mod II	residuals model I ~ poly(time,3) * treatment					
			Res.Df	Df.diff	val(F)	Pr(>F)	
	ANOVA table	(Intercept)	268	NA	NA	NA	
		poly(time,3)	265	3	10751.069	**0.004**	**
		treatment	263	2	4472.002	**0.004**	**
		poly(time,3):treatment	260	6	858.745	**0.004**	**
endobiota	mod I	community matrix ~ LSD + pop					
			Res.Df	Df.diff	Dev	Pr(>dev)	
	ANOVA table	(Intercept)	182	NA	NA	NA	
		LSD	181	1	16491.06	**0.002**	**
		population	175	6	40095.79	**0.006**	**
	mod II	residuals model I ~ poy(time,3) * treatment					
			Res.Df	Df.diff	val(F)	Pr(>F)	
	ANOVA table	(Intercept)	182	NA	NA	NA	
		poly(time,3)	179	3	3285.973	**0.004**	**
		treatment	177	2	2365.377	**0.004**	**
		poly(time,3):treatment	174	6	642.136	**0.004**	**

*Model structures are written in the syntax of the R package mvabund (Wang et al. 2012). The natural logarithm of the sequencing depth is abbreviated with LSD. p-values < 0.05 are displayed in bold and with stars (. < 0.1, * < 0.05, ** < 0.01 and *** < 0.001).*

Table S3. Statistical output of models fitted in this study. **(F)** β-diversity

model structure		~ poly(time,3) * treatment * (range + pop) + (1|population) + (1|individual)							
			Sum Sq	Mean Sq	NumDF	DenDF	F value	Pr(>F)	
epi - Bray-Curtis distances	ANOVA table	poly(time, 3)	0.351	0.117	3	1136.138	143.430	**8.25E-79**	***
		treatment	0.324	0.324	1	1113.915	396.503	**1.03E-75**	***
		range	0.015	0.015	1	13.051	17.880	**9.78E-04**	***
		pop	0.019	0.019	1	13.165	23.685	**2.97E-04**	***
		poly(time, 3):treatment	0.169	0.056	3	1133.769	69.187	**4.34E-41**	***
		poly(time, 3):range	0.110	0.037	3	1142.264	45.025	**1.67E-27**	***
		poly(time, 3):pop	0.051	0.017	3	1127.385	20.911	**3.46E-13**	***
		treatment:range	0.015	0.015	1	1118.174	18.787	**1.59E-05**	***
		treatment:pop	0.013	0.013	1	1108.682	16.525	**5.14E-05**	***
		poly(time, 3):treatment:range	0.062	0.021	3	1138.261	25.136	**9.50E-16**	***
		poly(time, 3):treatment:pop	0.013	0.004	3	1127.107	5.223	**0.001**	**
			Sum Sq	Mean Sq	NumDF	DenDF	F value	Pr(>F)	
endo - Bray-Curtis distances	ANOVA table	poly(time, 3)	0.069	0.023	3	504.683	17.770	**5.59E-11**	***
		treatment	0.012	0.012	1	485.917	9.160	**0.003**	**
		range	0.013	0.013	1	12.957	10.387	**0.007**	**
		pop	0.012	0.012	1	13.470	9.443	**0.009**	**
		poly(time, 3):treatment	0.034	0.011	3	489.090	8.854	**1.00E-05**	***
		poly(time, 3):range	0.076	0.025	3	513.655	19.624	**4.66E-12**	***
		poly(time, 3):pop	0.009	0.003	3	512.790	2.218	0.085	.
		treatment:range	0.044	0.044	1	493.691	33.842	**1.08E-08**	***
		treatment:pop	0.000	0.000	1	487.338	0.019	0.891	
		poly(time, 3):treatment:range	0.008	0.003	3	493.261	1.987	**0.115**	
		poly(time, 3):treatment:pop	0.002	0.001	3	487.688	0.556	0.645	
			Sum Sq	Mean Sq	NumDF	DenDF	F value	Pr(>F)	
epi - UniFrac distances	ANOVA table	poly(time, 3)	0.041	0.014	3	1151.684	68.894	**5.59E-41**	***
		treatment	0.009	0.009	1	1119.572	46.017	**1.89E-11**	***
		range	0.005	0.005	1	12.110	26.239	**2.45E-04**	***
		pop	0.004	0.004	1	12.639	19.273	**7.80E-04**	***
		poly(time, 3):treatment	0.012	0.004	3	1145.278	20.677	**4.74E-13**	***
		poly(time, 3):range	0.010	0.003	3	1158.093	16.583	**1.49E-10**	***
		poly(time, 3):pop	0.002	0.001	3	1139.416	2.717	**0.043**	*
		treatment:range	0.004	0.004	1	1123.783	21.144	**4.74E-06**	***
		treatment:pop	0.000	0.000	1	1111.731	1.825	0.177	
		poly(time, 3):treatment:range	0.003	0.001	3	1149.717	4.578	**0.003**	**
		poly(time, 3):treatment:pop	0.000	0.000	3	1136.229	0.732	0.533	
			Sum Sq	Mean Sq	NumDF	DenDF	F value	Pr(>F)	
endo - UniFrac distances	ANOVA table	poly(time, 3)	0.004	0.001	3	502.287	8.274	**2.21E-05**	***
		treatment	0.000	0.000	1	485.397	0.434	0.510	
		range	0.001	0.001	1	12.492	6.504	**0.025**	*
		pop	0.001	0.001	1	13.367	4.533	0.052	.
		poly(time, 3):treatment	0.003	0.001	3	487.402	4.896	**0.002**	**
		poly(time, 3):range	0.007	0.002	3	510.938	12.943	**3.68E-08**	***
		poly(time, 3):pop	0.001	0.000	3	509.883	2.080	0.102	
		treatment:range	0.000	0.000	1	493.047	1.144	0.285	
		treatment:pop	0.000	0.000	1	486.540	0.329	0.567	
		poly(time, 3):treatment:range	0.002	0.001	3	490.971	3.099	**0.027**	*
		poly(time, 3):treatment:pop	0.000	0.000	3	486.451	0.835	0.475	

*Model structures are written in the syntax of the R packages lme4 (Bates et al. 2015). The natural logarithm of the sequencing depth is abbreviated with LSD. P-values < 0.05 are displayed in bold and with stars (. < 0.1, * < 0.05, ** < 0.01 and *** < 0.001).*

Table S3. Statistical output of models fitted in this study. **(G)** Relative to field

model structure			~ poly(time,3) * individual * range + (1|individual)						
			Sum Sq	Mean Sq	NumDF	DenDF	F value	Pr(>F)	
epi - Bray-Curtis	ANOVA table	poly(time, 3)	0.047	0.016	3	464.259	20.233	**2.44E-12**	***
		range	0.009	0.009	1	115.694	11.234	**0.001**	**
		poly(time, 3):range	0.041	0.014	3	464.259	17.820	**5.80E-11**	***
			Sum Sq	Mean Sq	NumDF	DenDF	F value	Pr(>F)	
endo - Bray-Curtis	ANOVA table	poly(time, 3)	0.008	0.003	3	169.762	3.860	**0.011**	*
		range	0.000	0.000	1	77.735	0.341	0.561	
		poly(time, 3):range	0.006	0.002	3	169.762	2.658	**0.050**	*
			Sum Sq	Mean Sq	NumDF	DenDF	F value	Pr(>F)	
epi - UniFrac	ANOVA table	poly(time, 3)	0.007	0.002	3	470.157	9.711	**3.15E-06**	***
		range	0.006	0.006	1	116.181	27.309	**7.73E-07**	***
		poly(time, 3):range	0.014	0.005	3	470.157	20.035	**3.10E-12**	***
			Sum Sq	Mean Sq	NumDF	DenDF	F value	Pr(>F)	
endo - UniFrac	ANOVA table	poly(time, 3)	0.003	0.001	3	160.786	15.302	**8.33E-09**	***
		range	0.000	0.000	1	77.233	2.595	0.111	
		poly(time, 3):range	0.000	0.000	3	160.786	1.122	0.342	

*Model structures are written in the syntax of the R packages lme4 (Bates et al. 2015). The natural logarithm of the sequencing depth is abbreviated with LSD. P-values < 0.05 are displayed in bold and with stars (. < 0.1, * < 0.05, ** < 0.01 and *** < 0.001).*
